# Vasopressin mediates fructose-induced metabolic syndrome by activating the V1b receptor

**DOI:** 10.1172/jci.insight.140848

**Published:** 2021-01-11

**Authors:** Ana Andres-Hernando, Thomas J. Jensen, Masanari Kuwabara, David J. Orlicky, Christina Cicerchi, Nanxing Li, Carlos A. Roncal-Jimenez, Gabriela E. Garcia, Takuji Ishimoto, Paul S. Maclean, Petter Bjornstad, Laura Gabriela Sanchez-Lozada, Mehmet Kanbay, Takahiko Nakagawa, Richard J. Johnson, Miguel A. Lanaspa

**Affiliations:** 1 Division of Renal Diseases and Hypertension and; 2 Division of Endocrine, Diabetes, and Metabolism, University of Colorado Denver, Aurora, Colorado, USA.; 3 Department of Pathology, University of Colorado School of Medicine, Aurora, Colorado, USA.; 4 Department of Nephrology, Nagoya University Graduate School of Medicine, Nagoya, Japan.; 5 Division of Endocrinology, Metabolism and Diabetes, University of Colorado Denver Anschutz Medical Campus, Aurora, Colorado, USA.; 6 Department of Pediatrics, Section of Endocrinology, University of Colorado School of Medicine, Aurora, Colorado, USA.; 7 Department of Nephrology, Instituto Nacional de Cardiología-Ignacio Chavez, Mexico City, Mexico.; 8 Department of Medicine, Division of Nephrology, Koc University School of Medicine, Istanbul, Turkey.; 9 Department of Nephrology, Rakuwakai Otowa Hospital, Kyoto, Japan.

**Keywords:** Endocrinology, Metabolism, Carbohydrate metabolism, Obesity

## Abstract

Subjects with obesity frequently have elevated serum vasopressin levels, noted by measuring the stable analog, copeptin. Vasopressin acts primarily to reabsorb water via urinary concentration. However, fat is also a source of metabolic water, raising the possibility that vasopressin might have a role in fat accumulation. Fructose has also been reported to stimulate vasopressin. Here, we tested the hypothesis that fructose-induced metabolic syndrome is mediated by vasopressin. Orally administered fructose, glucose, or high-fructose corn syrup increased vasopressin (copeptin) concentrations and was mediated by fructokinase, an enzyme specific for fructose metabolism. Suppressing vasopressin with hydration both prevented and ameliorated fructose-induced metabolic syndrome. The vasopressin effects were mediated by the vasopressin 1b receptor (V1bR), as V1bR-KO mice were completely protected, whereas V1a-KO mice paradoxically showed worse metabolic syndrome. The mechanism is likely mediated in part by de novo expression of V1bR in the liver that amplifies fructokinase expression in response to fructose. Thus, our studies document a role for vasopressin in water conservation via the accumulation of fat as a source of metabolic water. Clinically, they also suggest that increased water intake may be a beneficial way to both prevent or treat metabolic syndrome.

## Graphical abstract

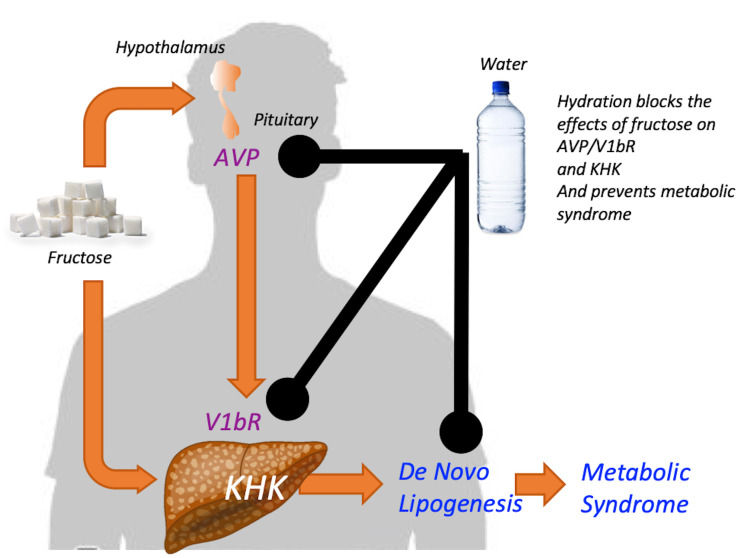

## Introduction

Sugar intake, especially from soft drinks, is strongly associated with the development of metabolic syndrome (1, 2) and may also increase cardiovascular mortality (3, 4). Therefore, reducing sugar intake is thought to improve metabolic syndrome (5–7). Experimental studies suggest that the fructose component in sugar is the primary culprit (8) and that fructose induces its effects by shifting energy production to energy storage (9–12). High-glucose ingestion can also cause metabolic syndrome but primarily through the conversion of glucose to fructose in the liver via activation of the polyol (aldose reductase) pathway (13). Fructose has a unique metabolism in which the first enzyme, fructokinase C (ketohexokinase C [KHK-C]), metabolizes fructose with consumption of ATP so rapidly that a transient depletion of intracellular phosphate and ATP occurs (14, 15). Consistently, KHK-C is thought to be the main driver of fructose-induced metabolic syndrome and is expressed in those organs involved in metabolizing the majority of dietary fructose, including liver and small intestine. KHK-C is also present in selected areas of the kidney cortex, pancreas and brain. Another KHK isoform, namely KHK-A, is more ubiquitously expressed but has a lower affinity for fructose and as such its role in metabolic syndrome is thought to be substantially weaker.

A striking observation is that participants with metabolic syndrome, and/or related conditions, such as nonalcoholic fatty liver disease (NAFLD), often have elevated concentrations of vasopressin, as determined by measuring its stable analog copeptin (16–19), a stable peptide derived from the precursor of vasopressin (20). Elevated copeptin can also increase the risk of obesity and diabetes (17, 21). Furthermore, there is also experimental evidence documenting that chronic vasopressin infusion can worsen glycemic control in lean and obese Zucker rats; and in obese rats, vasopressin promoted the development of insulin resistance; whereas hydration-mediated reduction in vasopressin concentrations attenuated liver steatosis (22).

Fructose intake can modulate vasopressin levels. For example, giving intravenous fructose but not iso-osmolar glucose stimulates vasopressin secretion in humans (23). Fructose also stimulates vasopressin synthesis when added directly to hypothalamic explants of mice; this was found to be mediated through fructokinase, a key enzyme in fructose metabolism (24). We have also reported that both acute and chronic dehydration, by increasing osmolality, can induce activation of aldose reductase in the hypothalamus, leading to local fructose production that drives some of the vasopressin response (24). Others have documented production of fructose in the brain from polyol-dependent conversion of glucose to fructose (25). More recently, soft drinks have been reported to increase vasopressin concentrations in both laboratory animals and humans following heat stress (26, 27).

To date, however, the role of vasopressin in sugar-induced metabolic syndrome is not known. Here we tested the hypothesis that vasopressin is a mediator of fructose-induced obesity and diabetes.

## Results

### Fructose metabolism by fructokinase promoted vasopressin production during the development of metabolic syndrome.

**Figure 1:**
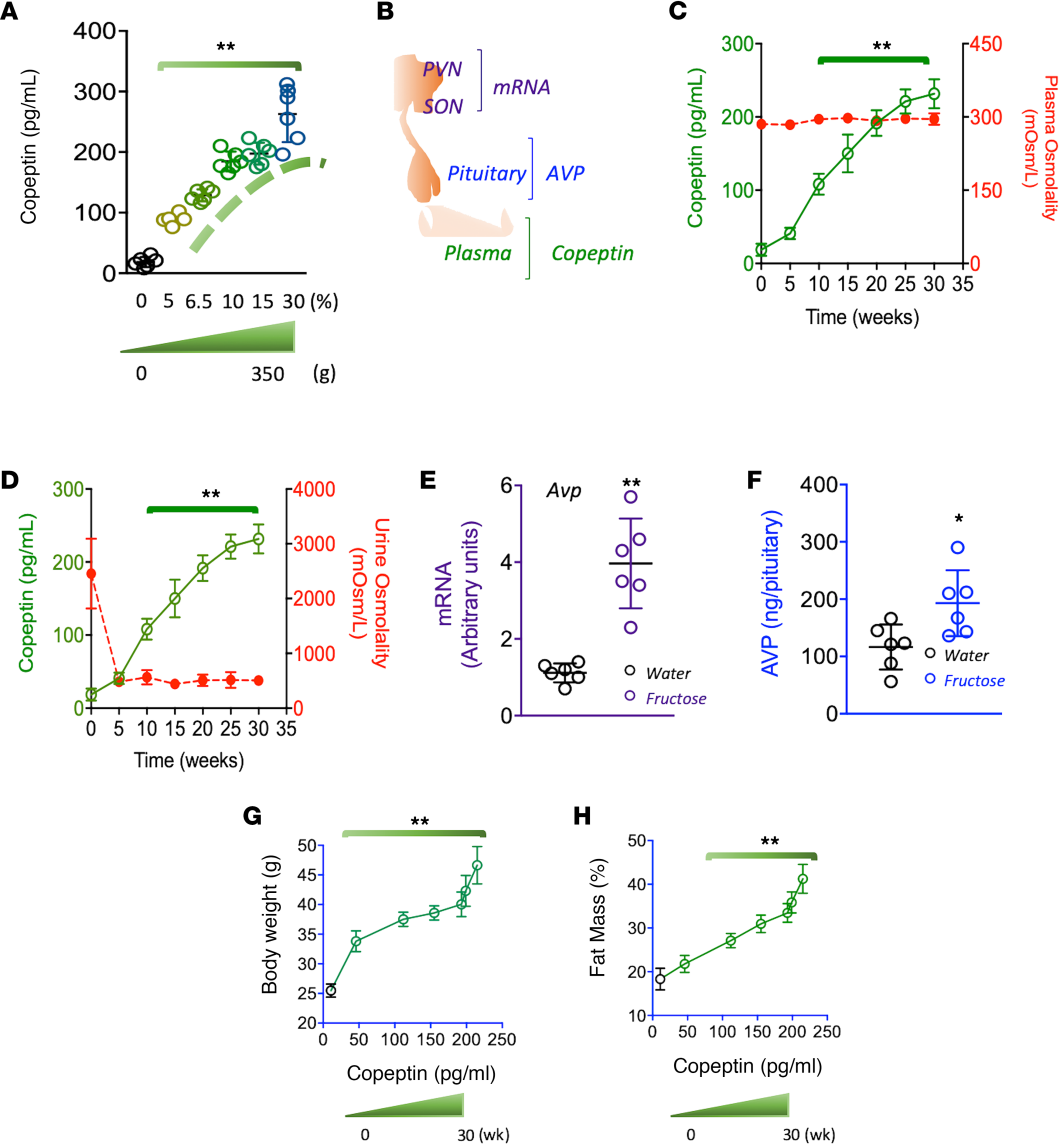
Fructose promotes vasopressin production and secretion during the development of metabolic syndrome. (**A**) Serum copeptin levels in mice receiving fructose solutions (from 0 to 30%) for 30 weeks. Cumulative fructose intakes varied from 0 to 350 g/mouse. (**B**) Schematic depicting the areas for vasopressin production (hypothalamic nuclei), accumulation (posterior pituitary), and secretion (serum). (**C**) Serum copeptin and osmolality levels in mice receiving a 10% fructose solution for 30 weeks. (**D**) Serum copeptin levels and urinary osmolality in mice receiving a 10% fructose solution for 30 weeks. (**E**) Hypothalamic mRNA levels of vasopressin in mice receiving water or a 10% fructose solution for 30 weeks. (**F**) Vasopressin levels in pituitary of mice receiving a 10% fructose solution for 30 weeks. (**G**) 5-week body weight/copeptin correlations in mice receiving a 10% fructose solution for 30 weeks. (**H**) Adiposity (% of fat mass) in mice receiving a 10% fructose solution for 30 weeks. The data in **A** and **C–H** are presented as the mean ± SD and analyzed by 1-way ANOVA with Tukey’s post hoc analysis. *P < 0.05, **P < 0.01. *n* = 6 mice per group. PVN, paraventricular nuclei; SON*,* supraoptic nuclei.

We first evaluated whether vasopressin concentrations in serum, hypothalamus, and posterior pituitary were affected by fructose in mice (Figure 1). We and others have previously demonstrated that fructose in the drinking water is a potent stimulator of metabolic syndrome in mice (Supplemental Table 1; supplemental material available online with this article; https://doi.org/10.1172/jci.insight.140848DS1). Interestingly, fructose solutions provided in the drinking water showed a dose (Figure 1A) and time-dependent response (Figure 1, C and D) in serum copeptin concentrations over a 30-week period without modifying serum or urine osmolality. Furthermore, serum copeptin increased after administration of 6.5% fructose, which is comparable to the percent of fructose in the average soft drink. Fructose (10%) in the drinking water was also found to increase hypothalamic expression of vasopressin mRNA (Figure 1E) and increased vasopressin accumulation in the pituitary (Figure 1F). Of interest, the observed fructose-induced temporal increase in copeptin correlated with metabolic markers, including body mass and adiposity (Figure 1, G and H), suggestive of a potential role for vasopressin as a mediator of metabolic syndrome induced by fructose.

**Figure 2:**
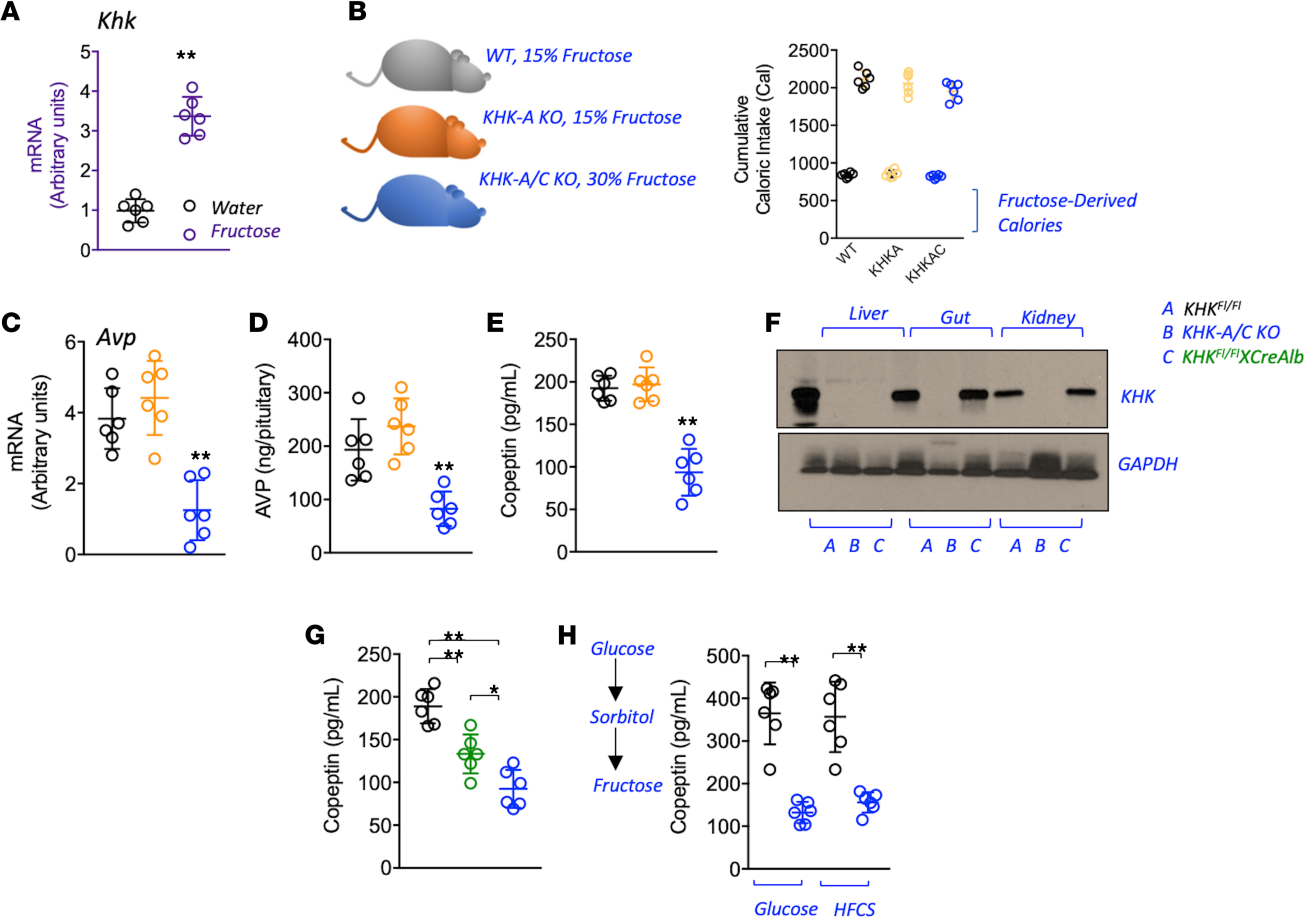
Fructose metabolism via fructokinase is necessary for vasopressin production and secretion. (**A**) Hypothalamic mRNA levels of fructokinase (KHK) in mice receiving water or a 10% fructose solution for 30 weeks. (**B**) Cumulative total and fructose-derived caloric intake in WT (black), KHK-A–KO (orange), and KHK-A/C–KO (blue) mice receiving equal amounts of fructose for 30 weeks. (**C**) Hypothalamic mRNA levels of vasopressin in WT, KHK-A–KO, and KHK-A/C–KO mice receiving equal amounts of fructose for 30 weeks. (**D**) Vasopressin levels in pituitary of WT, KHK-A–KO, and KHK-A/C–KO mice receiving equal amounts of fructose for 30 weeks. (**E**) Serum copeptin levels in WT, KHK-A–KO, and KHK-A/C–KO mice receiving equal amounts of fructose for 30 weeks. (**F**) Representative Western blot (*n* = 3 total blots) for KHK and actin in liver, gut, and kidney tissues from WT (black), KHK-A/C–KO (blue), and liver-specific KHK-A/C–KO mice (*KHK^Fl/Fl^XCreAlb*, green). (**G**) Serum copeptin levels in WT, KHK-A/C–KO, and liver-specific KHK-A/C–KO mice receiving equal amounts of fructose for 30 weeks. (**H**) Serum copeptin levels in WT and KHK-A/C–KO mice receiving glucose (10%) or HFCS (10%) solutions for 30 weeks. The data in **A–E** and **G** and **H** are presented as the mean ± SD and analyzed by 1-way ANOVA with Tukey’s post hoc analysis. *P < 0.05, **P < 0.01. *n* = 6 mice per group. See also Supplemental Table 1 and Supplemental Table 2. KHK, ketohexokinase; KHK-A, A isoform of KHK; KHK-A/C, both A and C isoforms of KHK; HFCS, high-fructose corn syrup.

After establishing a direct relationship between fructose intake and vasopressin concentrations, we next evaluated whether this correlation required metabolism of the fructose via fructokinase (KHK). Of interest, KHK expression is markedly higher in the hypothalamus of mice exposed to fructose (Figure 2A). To characterize the importance of KHK in fructose-mediated vasopressin activation, we provided equivalent amounts of fructose in the drinking water to WT mice or mice lacking the A isoform of KHK (systemic KHK-A KO) or both the A and C isoforms (systemic KHK-A/C KO). To this end, and because KHK-A/C–KO mice do not prefer fructose, whereas WT or KHK-A–KO mice love it, we provided 30% fructose in the drinking water of KHK-A/C–KO mice, but only 15% fructose in the drinking water of WT and KHK-A–KO mice (Figure 2B) as previously described (28). After treatment, KHK-A/C–KO mice demonstrated markedly lower hypothalamic vasopressin mRNA, vasopressin protein in the pituitary, and serum copeptin compared with WT and KHK-A–KO mice on fructose (Figure 2, C–E, and Supplemental Table 1). To further understand the mechanism and clinical relevance of these findings and considering that the blockade of hepatic KHK is sufficient to prevent metabolic syndrome induced by fructose in mice, we then determined if the vasopressin response was mediated by hepatic fructose metabolism by using liver-specific KHK-A/C–KO mice (Figure 2F) (29). Of interest, liver-specific KHK-A/C–KO mice mounted a significantly lower copeptin response to fructose than WT mice, although it remained higher than that observed in the systemic KHK-A/C–KO mice (Figure 2G and Supplemental Table 2), suggesting that the liver plays a partial role in regulating vasopressin in response to fructose. We also evaluated whether drinking water containing high-fructose corn syrup (HFCS), or glucose (which can be converted to fructose in the body; ref. 13) stimulated copeptin. As shown in Figure 2H, both HFCS and glucose alone stimulated copeptin in WT but not KHK-A/C–KO mice, thus supporting an important but deleterious role of endogenous fructose production and metabolism in the sugar-dependent vasopressin response.

### Suppressing vasopressin by increasing water intake prevented and treated fructose-induced metabolic syndrome.

We next sought the significance of the vasopressin response by attempting to block its expression with hydration. Indeed, there are pilot studies in humans suggesting that increasing water intake by 1.5 L/d for 6 weeks can reduce copeptin concentrations in humans in association with a significant reduction in fasting serum glucose (30). To increase water intake, we made hydrogels by mixing 3 mL of water per gram of powdered chow using 4% agar, similar to the method used by Bouby et al. (31). We first confirmed that the use of hydrogels could increase total water intake in normal WT mice and that this was associated with a reduction in urine osmolality and copeptin concentrations (Supplemental Figure 1).

**Figure 3:**
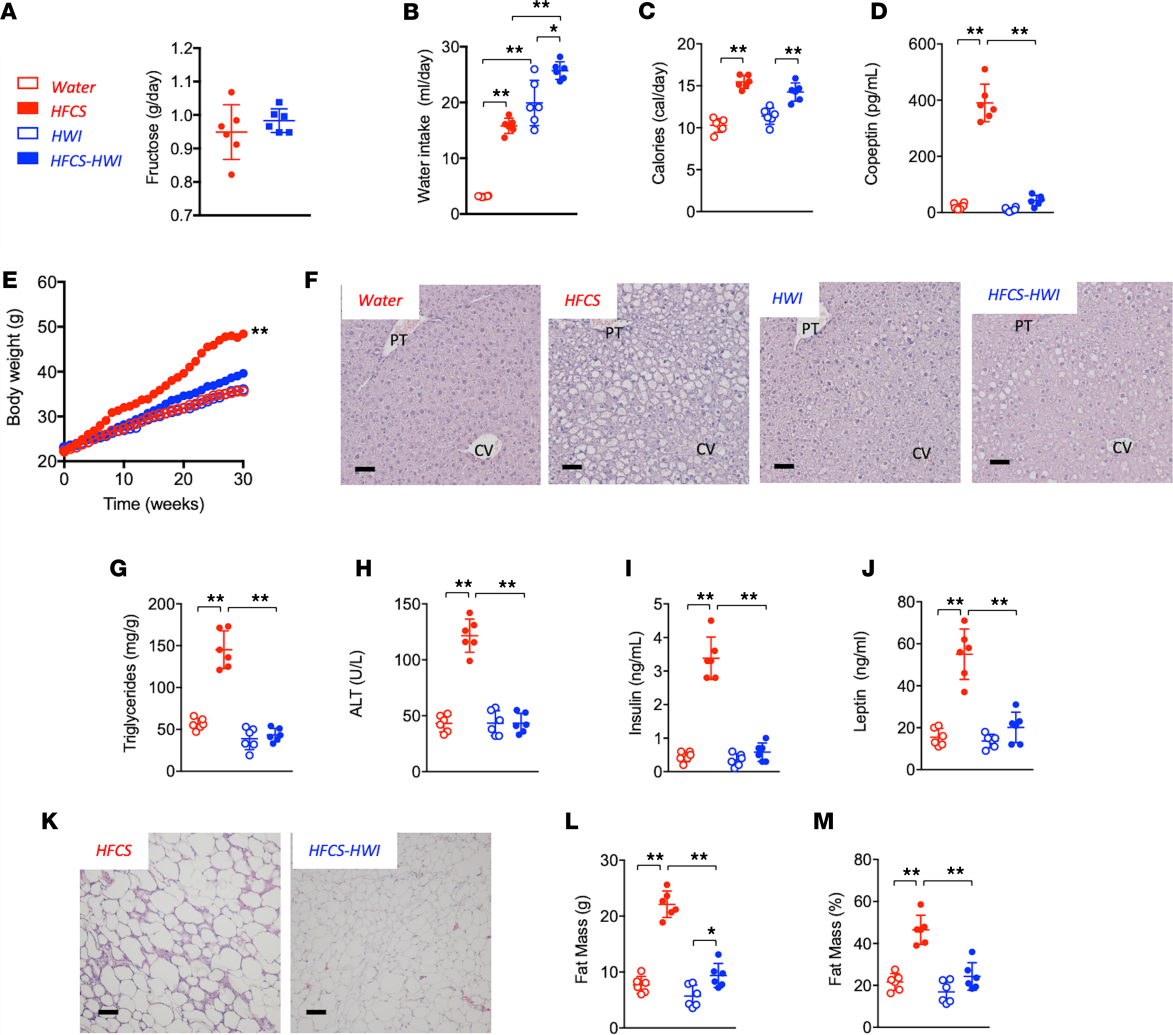
Lowering vasopressin protects against sugar-induced metabolic syndrome. (**A**) Average daily fructose intake (g/day) in the drinking water in WT mice receiving HFCS (red) alone or in combination with hydrogels (HFCS-HWI, blue) for 30 weeks. (**B**) Average daily total water intake (mL/day) in WT mice control (water, red clear bars), receiving HFCS (red solid bars), hydrogels (HWI, blue clear bars), or HFCS in combination with hydrogels (HFCS-HWI, blue solid bars) for 30 weeks. (**C**) Average daily caloric intake water, HFCS, HWI, and HFCS-HWI groups. (**D**) Serum copeptin levels at 30 weeks in water, HFCS, HWI, and HFCS-HWI groups. (**E**) Weekly body weight gain in water, HFCS, HWI, and HFCS-HWI groups. (**F**) Representative H&E images from livers of mice (*n* > 10 images per animal) of the same groups as in **B** at 30 weeks. Size bars: 50 μM. (**G**) Liver triglycerides (normalized to protein levels) at 30 weeks in water, HFCS, HWI, and HFCS-HWI groups. (**H**) Serum ALT levels at 30 weeks in water, HFCS, HWI, and HFCS-HWI groups. (**I**) Serum insulin levels at 30 weeks in water, HFCS, HWI, and HFCS-HWI groups. (**J**) Serum leptin levels at 30 weeks in water, HFCS, HWI, and HFCS-HWI groups. (**K**) Representative H&E images from epididymal adipose tissue of mice (*n* > 10 images per animal) on HFCS or HFCS-HWI groups. Size bars: 50 μM. (**L**) Total fat mass (g) at 30 weeks in water, HFCS, HWI, and HFCS-HWI groups. (**M**) Fat mass to total body weight percentage at 30 weeks in water, HFCS, HWI, and HFCS-HWI groups. The data in **A–E, G–J**, and **L** and **M** are presented as the mean ± SD and analyzed by 1-way ANOVA with Tukey’s post hoc analysis except for **A**, which was analyzed by a 2-tailed *t* test. *P < 0.05, **P < 0.01. *n* = 6 mice per group. See also Supplemental Figure 1 and Supplemental Table 3. HFCS, high-fructose corn syrup; HWI, high water intake; PT, portal triad; CV, central vein; ALT, alanine aminotransferase.

We queried whether increasing water intake could suppress the induction of metabolic syndrome mediated by HFCS (15% in the water, i.e., 9% fructose and 6% glucose) for 30 weeks. We administered HFCS because it is more representative of the human diet than fructose alone. Control groups included WT mice receiving normal water intake (NWI) or high water intake (HWI) in the absence of HFCS. As shown in Figure 3 and Supplemental Table 3, fructose intake was similar between HFCS-NWI mice and HFCS-HWI mice (Figure 3A), but total water intake was significantly increased in the HWI groups (Figure 3B). Although the caloric intake remained high in the HFCS-HWI group (Figure 3C), there was a remarkable lowering of serum copeptin (Figure 3D) in association with lower body weight (Figure 3E), fatty liver (Figure 3, F–H), hyperinsulinemia and hyperleptinemia (Figure 3, I and J), adipocyte inflammatory changes (Figure 3K), and fat mass and percentage (Figure 3, L and M). For reference, additional metabolic parameters are shown in Supplemental Table 3.

**Figure 4:**
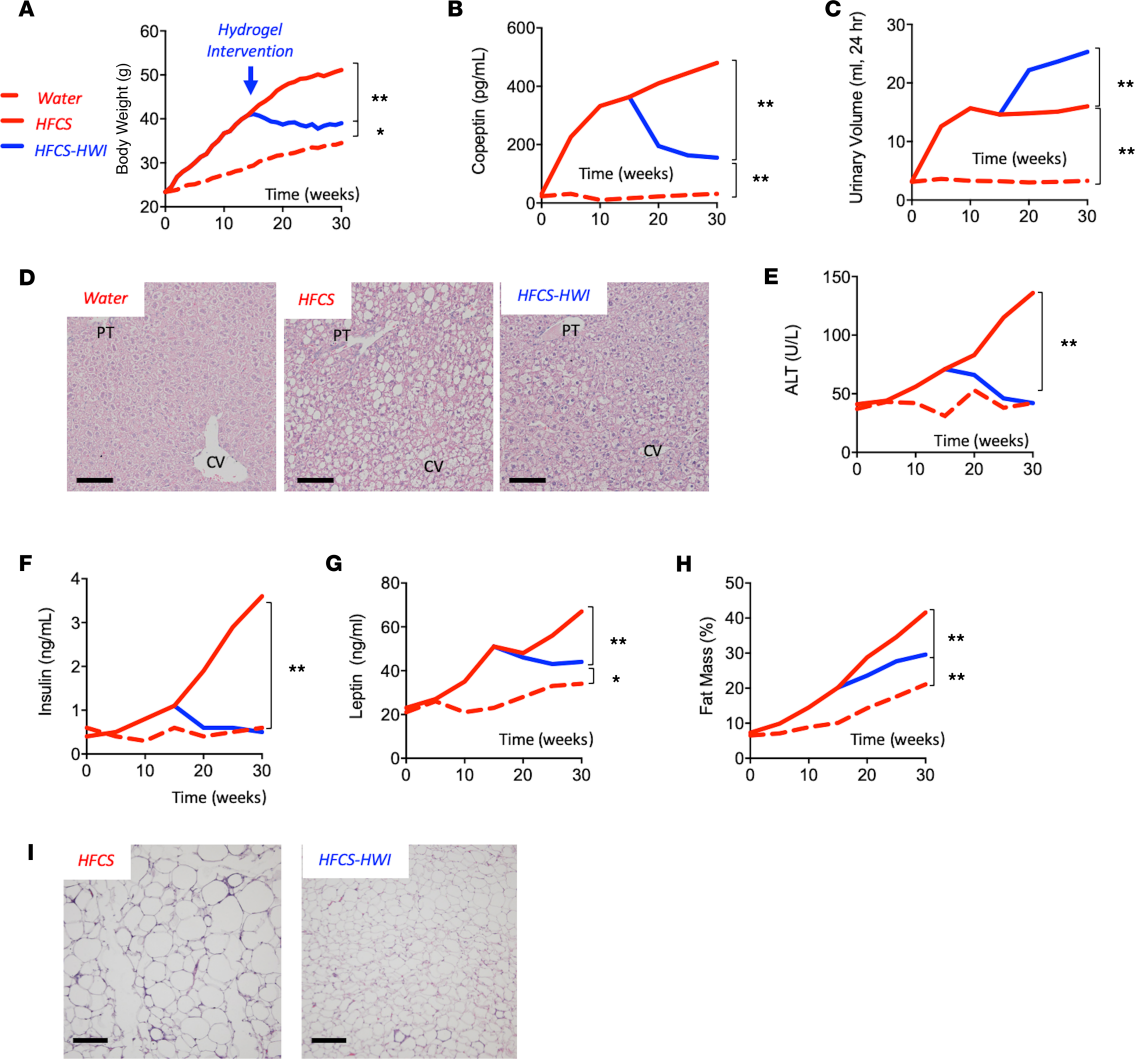
Lowering vasopressin as a therapeutic intervention in mice with sugar-induced metabolic syndrome. (**A**) Weekly body weight gain in mice receiving water (red-dashed line) or HFCS (red solid line) for 30 weeks. At week 15 a subgroup of HFCS started the intervention with hydrogels (HFCS-HWI, blue solid line). (**B**) 30-week serum copeptin levels in water, HFCS, and HFCS-HWI groups. (**C**) 30-week urinary volume excretion (mL urine/24 hour) in water, HFCS, and HFCS-HWI groups. (**D**) Representative H&E images from livers of mice (*n* > 10 images per animal) of the same groups as in **A** at 30 weeks. Size bars: 50 μM. (**E**) 30-week serum ALT levels in water, HFCS, and HFCS-HWI groups. (**F**) 30-week serum Insulin levels in water, HFCS, and HFCS-HWI groups. (**G**) 30-week serum leptin levels in water, HFCS, and HFCS-HWI groups. (**H**) 30-week fat mass to total body weight percentage in water, HFCS, and HFCS-HWI groups. (**I**) Representative H&E images from epididymal adipose tissue of mice (*n* > 10 images per animal) on HFCS or HFCS-HWI groups. Size bars: 50 μM. The data in **A–C** and **E–H** are presented as the mean and analyzed by 1-way ANOVA with Tukey’s post hoc. The data for **A** were collected and analyzed weekly, whereas the data for **B** and **C** and **E–H** were collected and analyzed every 5 weeks. *P < 0.05, **P < 0.01. *n* = 6 mice per group. See also Supplemental Figure 1 and Supplemental Table 4. HFCS, high-fructose corn syrup; HWI, high water intake; PT, portal triad; CV, central vein; ALT, alanine aminotransferase.

We evaluated whether lowering serum vasopressin levels could be a useful strategy to ameliorate the metabolic effects of fructose in already obese mice. To this end, we exposed mice to HFCS for 15 weeks, at which time we randomized the mice into 2 groups, an HWI group maintained on HFCS plus hydrogels and a NWI group maintained on HFCS and regular chow. After an additional 15 weeks, the mice were sacrificed, and the features of metabolic syndrome noted above were analyzed in these mice. As shown in Figure 4 and Supplemental Table 4, body weight gain (Figure 4A) was markedly attenuated and serum copeptin concentrations fell (Figure 4B) in HFCS-HWI mice compared with HFCS-NWI mice in association with increased urine volumes (Figure 4C). This was also associated with an improvement in fatty liver (Figure 4, D and E), hyperinsulinemia and hyperleptinemia (Figure 4, F and G), fat mass, and adipose inflammation (Figure 4, H and I).

### Opposing effects of vasopressin receptors V1a and V1b in fructose-induced metabolic syndrome.

**Figure 5:**
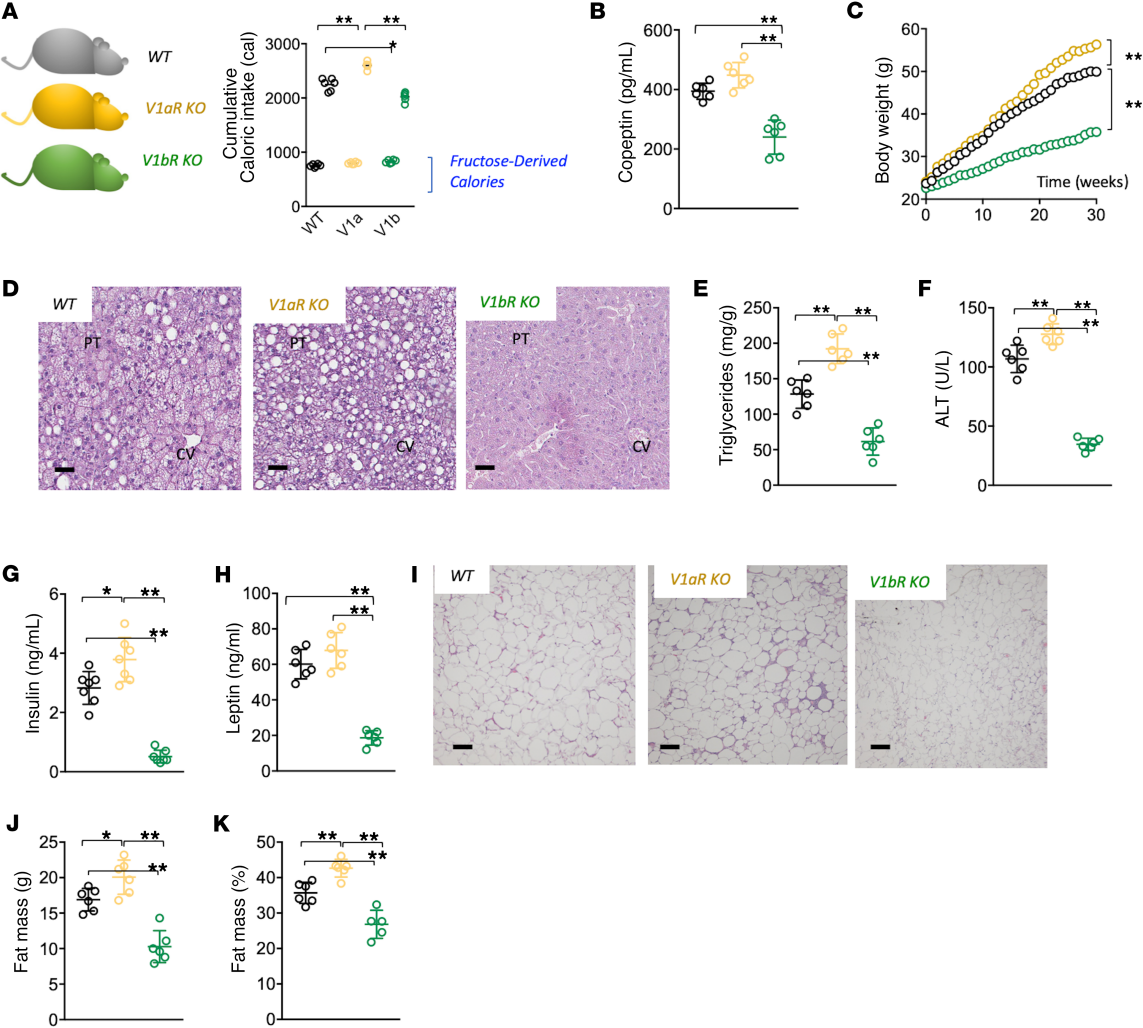
Opposing effects of vasopressin receptors in fructose-induced metabolic syndrome. (**A**) 30-week cumulative total and fructose-derived caloric intake in WT (black), V1aR-KO (ochre), and V1bR-KO (green) mice on 10% fructose. (**B**) Serum copeptin levels in WT, V1aR-KO, and V1bR-KO mice receiving a 10% fructose solution for 30 weeks. (**C**) Weekly body weight gain in WT, V1aR-KO, and V1bR-KO mice receiving a 10% fructose solution for 30 weeks. (**D**) Representative H&E images from livers of mice (*n* > 10 images per animal) of the same groups as in **A** at 30 weeks. Size bars: 50 μM. (**E**) Liver triglycerides (normalized to protein levels) at 30 weeks in WT, V1aR-KO, and V1bR-KO mice receiving a 10% fructose solution. (**F**) Serum ALT levels at 30 weeks in WT, V1aR-KO, and V1bR-KO mice receiving a 10% fructose solution. (**G**) Serum insulin levels at 30 weeks in WT, V1aR-KO, and V1bR-KO mice receiving a 10% fructose solution. (**H**) Serum leptin levels at 30 weeks in WT, V1aR-KO, and V1bR-KO mice receiving a 10% fructose solution. (**I**) Representative H&E images from epididymal adipose tissue of mice (*n* > 10 images per animal) of the same groups as in **A** at 30 weeks. Size bars: 50 μM. (**J**) Total fat mass (g) at 30 weeks in WT, V1aR-KO, and V1bR-KO mice receiving a 10% fructose solution. (**K**) Fat mass to total body weight percentage at 30 weeks in WT, V1aR-KO, and V1bR-KO mice receiving a 10% fructose solution. The data in **A–C**, **E–H**, and **J** and **K** are presented as the mean ± SD and analyzed by 1-way ANOVA with Tukey’s post hoc analysis. *P < 0.05, **P < 0.01. *n* = 6 mice per group. See also Supplemental Table 5. V1aR, vasopressin 1a receptor; V1bR, vasopressin 1b receptor; PT, portal triad; CV, central vein; ALT, alanine aminotransferase.

Whereas the antidiuretic effects of vasopressin are mediated by the V2 receptor expressed in the collecting duct of the kidney, the metabolic effects of fructose are primarily mediated by the V1a and V1b receptors (32). To evaluate the role of these receptors, we administered fructose solutions (10%) or regular water to WT, V1aR-KO, and V1bR-KO mice for 30 weeks (Figure 5 and Supplemental Table 5). Total caloric intake was substantially higher in V1aR-KO mice than WT or V1bR-KO mice with no significant differences in fructose-derived calories between the strains (Figure 5A). All strains on fructose demonstrated a marked elevation in serum copeptin and vasopressin production compared with control animals receiving regular water (Supplemental Table 5). However, it is important to note that serum copeptin levels in fructose-fed V1bR-KO mice were significantly lower than WT or V1aR-KO mice (Figure 5B), suggestive of a potential regulation of KHK expression by vasopressin via the V1b receptor.

Consistent with lower vasopressin concentrations, V1bR-KO mice demonstrated reduced features of metabolic syndrome. On the other hand, V1aR-KO mice had higher serum copeptin concentrations and in general demonstrated a worse metabolic phenotype compared with WT mice (Figure 5 and Supplemental Table 5).

Thus, these studies showed that the V1bR plays a major role in mediating fructose-induced metabolic syndrome.

### Hepatic vasopressin V1b receptor drove metabolic syndrome by stimulating fructokinase and liver fructose metabolism.

**Figure 6:**
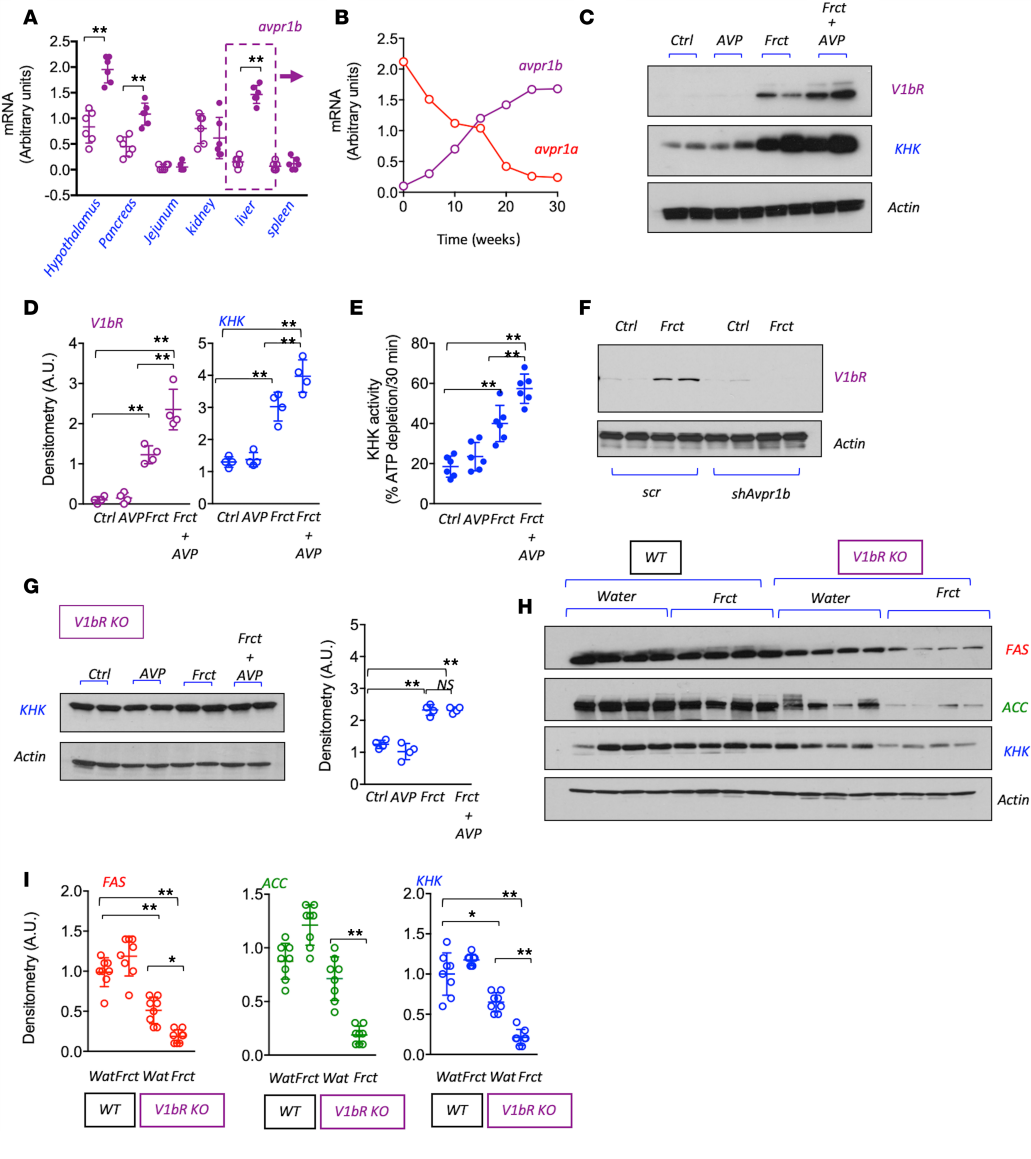
Hepatic V1bR potentiates the lipogenic effects of fructose. (**A**) Transcriptional levels of the *avpr1b* in hypothalamus, pancreas, jejunum, kidney, liver, and spleen of WT mice on water Ctrl (clear purple bars) or receiving a 10% Frct solution for 30 weeks (solid purple bars). (**B**) Transcriptional levels of the *avpr1a* (red line) and the *avpr1b* (purple line) in liver of WT mice receiving a 10% Frct solution for 30 weeks. (**C** and **D**) Representative Western blot and densitometry (*n* = 2 total blots) for the V1bR, fructokinase (KHK), and actin in human HepG2 cells Ctrl or exposed to AVP (250 nM), Frct (10 mM), or a combination of Frct plus AVP for 5 days. (**E**) KHK activity in HepG2 lysates from Ctrl, AVP, Frct, and Frct plus AVP cells. (**F**) Representative Western blot and densitometry (*n* = 2 total blots) for V1bR and actin in HepG2 transduced with noncodifying shRNA (*scr*) or shRNA against avpr1b (*shAvpr1b*) at baseline or a Frct (10 mM) exposure. (**G**) Representative Western blot and densitometry (*n* = 2 total blots) for KHK and actin in Ctrl, AVP, Frct, and Frct plus AVP HepG2 cells stably silenced for V1bR expression. (**H** and **I**) Representative Western blot (*n* = 2 total blots) and densitometry for KHK, actin, and lipogenic enzymes FAS and ACC in the liver of WT and V1bR-KO mice on water Ctrl or receiving a 10% Frct solution for 30 weeks. The data in **A** and **C–E** are presented as the mean ± SD and analyzed by 1-way ANOVA with Tukey’s post hoc analysis. *P < 0.05, **P < 0.01. For **A** and **B** and **E**, *n* = 6 mice per group. For **C**–**E**, *n* = 2 independent cultured plates. V1bR, vasopressin 1b receptor; *avpr1b*, vasopressin 1b receptor gene; *avpr1a*, vasopressin 1a receptor gene; KHK, ketohexokinase; Ctrl, control; AVP, vasopressin; Frct, fructose; *scr*, scramble; FAS, fatty acid synthase; ACC, acetyl-CoA carboxylase.

We next evaluated the sites of expression of V1bR and found that fructose administration markedly induced V1bR mRNA expression in the hypothalamus, pancreas, and liver (Figure 6A). Whereas V1bR is not normally expressed in liver, it has been reported to be induced in obese mice (33). Indeed, we found that V1bR (*avpr1b*) mRNA expression was induced over the 30-week period and with a parallel decrease in V1aR (*avpr1a*) mRNA levels (Figure 6B).

Previous studies by our group propose a key role for hepatic KHK in mediating metabolic syndrome induced by fructose (29, 34). This led us to hypothesize that V1bR might be influencing hepatic KHK expression and activity. To evaluate this, we examined the effect of vasopressin with or without fructose on human HegG2 cells expression of the V1bR and KHK. As shown in Figure 6, C–D, under normal conditions or after incubation with vasopressin alone, V1bR is not expressed in HepG2 cells, with minimal KHK expression. However, the incubation with fructose upregulated both V1bR and KHK expressions, which was markedly augmented when fructose and vasopressin were provided in combination. Consistent with higher expression, KHK activity was greater by the combination of fructose and vasopressin (Figure 6E). Furthermore, in HepG2 cells stably silenced for V1bR (Figure 6F), the synergistic effect of vasopressin and fructose to enhance expression of KHK was markedly prevented (Figure 6G), indicating that the expression of V1bR in the liver is necessary to stimulate KHK expression in response to fructose. Consistent with these findings, levels of KHK, as well as expression of lipogenic enzymes fatty acid synthase (FAS) and acetyl-CoA carboxylase (ACC), were inhibited in the livers of V1bR-KO mice (Figure 6, H–I).

## Discussion

Elevated concentrations of copeptin predict the development of metabolic syndrome (17, 21). Here we investigated whether the underlying mechanism may involve fructose and whether vasopressin might have a causal role in sugar-induced metabolic syndrome. The major findings of the study were that oral fructose-stimulated vasopressin levels and suppression of vasopressin with water prevented and attenuated metabolic syndrome in mice. We also found that these mechanisms were mediated by activation of the V1b receptor and that the effects were likely driven by upregulating fructokinase activity.

WT mice fed fructose orally exhibited higher serum vasopressin concentrations. This was observed with doses of fructose (6.5% in the drinking water) that are equivalent to humans in the upper quintile of sugar intake (4). The data are consistent with studies showing that the acute administration of intravenous fructose but not glucose can stimulate vasopressin in humans (23), as well as with recent studies showing heat-stressed humans hydrated with soft drinks have increased copeptin levels not observed in those equally hydrated with water (27). We found that the mechanism is not mediated by osmolality but is dependent on KHK (24), and indeed in our study, the KHK-A/C–KO mice on fructose did not have an elevated vasopressin concentration. Similarly, the rise in copeptin in our fructose-fed mice was not secondary to the increase in weight or development of metabolic syndrome because V1b-KO mice on fructose also showed high-copeptin concentrations despite being protected from metabolic syndrome. The acute effects of fructose on vasopressin observed in humans and animals also suggest it is independent of obesity or metabolic syndrome (23, 24, 27). Interestingly, the data showing that vasopressin levels are lower in the liver-specific KHK-KO mice document that the liver also plays a partial role in modulating vasopressin levels in addition to the hypothalamus.

We next evaluated the function of vasopressin in metabolic syndrome by suppressing vasopressin levels with increased water intake. For these studies, we used HFCS because this is a major sweetener used in western societies. Here we found that increasing water intake could suppress the development of the metabolic syndrome and, very importantly, it could also ameliorate established metabolic syndrome. In this regard, Taveau et al. have also reported that water loading could reduce fatty liver in the obese Zucker rat (22). Although the relative increase in water intake was substantial, a recent clinical pilot trial showed that doubling water intake could reduce copeptin concentrations and fasting glucose levels in humans (30). Hence, the clinical implication of this finding could be of interest for people with obesity.

We also investigated the mechanism(s) by which vasopressin might cause metabolic syndrome. The major finding was that the metabolic syndrome was mediated by activation of the V1b and not the V1a receptor. Although activation of the V1a receptor on the liver stimulates gluconeogenesis in the liver, V1a receptor expression has been reported to be low in people with NAFLD (35). We also found that V1aR expression fell to low levels in our model of fructose-induced fatty liver and metabolic syndrome. Importantly, however, we found that V1bR mRNA was induced de novo in fructose-induced fatty liver and upregulated at other sites, including the pancreas and hypothalamus. Unfortunately, no validated V1bR antibodies for mice were available and the confirmation of its silencing relies on genotyping and QPCR. A similar issue relates to the selectivity of V1bR antibodies for Western blot in human samples. Fortunately, the lot analyzed from a commercial antibody (see Methods) confirmed its efficiency in human samples, as shown in Figure 6E.

**Figure 7:**
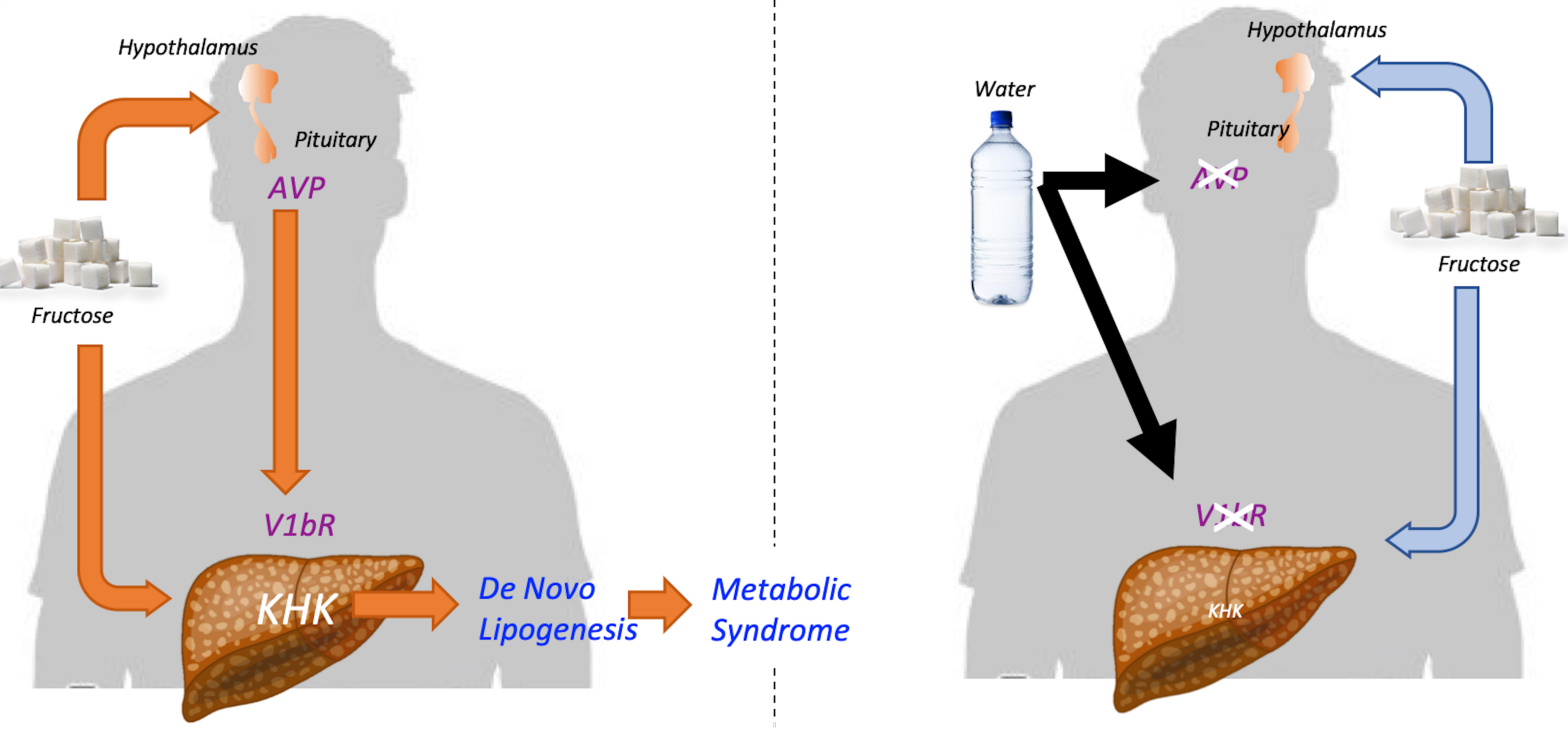
Proposed mechanism for the interplay between fructose and vasopressin in metabolic syndrome. (Left side, orange lines) Fructose stimulates both the expression of fructokinase (KHK) and the induction of the V1bR in the liver. Fructose metabolism in both liver and hypothalamus stimulates the production and secretion of AVP. The actions of AVP on hepatic V1bR potentiate the metabolic effects of fructose on the expression of KHK and lipogenic enzymes FAS and ACC. As a result, AVP and fructose promote fatty liver, adiposity, and body weight gain during the development and progression of metabolic syndrome. (Right side, blue lines) Hydration and other strategies directed to lower circulating AVP levels would decrease the hepatic expression of both V1bR and KHK in response to fructose. As a consequence, less fructose would be metabolized into fat, thus limiting the progression of metabolic syndrome. V1bR, vasopressin 1b receptor; AVP, vasopressin; KHK, ketohexokinase; FAS, fatty acid synthase; ACC, acetyl-CoA carboxylase.

We also investigated whether the hepatic V1bR might mediate the metabolic effects of fructose. Fructose is known to increase KHK expression and activity (36), and in our studies, we found this was amplified by the presence of vasopressin. Moreover, by using human HepG2 cells, we were able to show that this amplification system was mediated by direct binding of vasopressin to V1bR on hepatocytes, as knocking down V1bR prevented the amplification. Importantly, these studies do not rule out other known actions related to the activation of V1bR, such as its effects via glucagon or ACTH. A summary of the proposed mechanism for the interplay of fructose with vasopressin is shown in Figure 7.

The physiological significance of our study is that vasopressin, beyond having a role in water conservation via urinary concentration, may also increase fat production, possibly as a mechanism for storing metabolic water. In essence, fat is a source of water since the metabolism of fat (and glycogen) produces “metabolic” water. Many animals that have reduced water availability have high vasopressin levels and high visceral fat content, including desert and marine mammals (37). Vasopressin may also reduce water losses by other mechanisms, including via activation of V2 receptors in the lungs, or by the hypothermic effects of V1bR activation (37). Hence, vasopressin might be considered a survival hormone that stimulates fat accumulation as a means for preserving and storing energy and water.

The clinical significance of this work is that it may encourage studies to evaluate whether simple increases in water intake may effectively mitigate obesity and metabolic syndrome (30, 38). It may also be important to evaluate the balance of salt and water intake because vasopressin increases by an elevation in serum osmolality, which in turn activates aldose reductase in the liver and stimulates endogenous fructose production (39). Recently, our group found that a high-salt diet can, by raising serum osmolality, induce fructose production and metabolic syndrome (39), and there is increasing evidence that high-salt diets increase the risk of obesity and metabolic syndrome in humans (39–43). Indeed, even the acute blood pressure effects of salt may be mediated by changes in osmolality (44), and hyperosmolality may be a better predictor for hypertension than sodium intake alone (45). Hence, a balanced approach that increases water and reduces salt intake might provide an additional yet simple approach to treating the metabolic syndrome and obesity.

Limitations of the study include the fact that although we characterized the response of human hepatocytes to vasopressin and fructose, most of the experiments in this study involved mice and not humans. In addition, the observed complete blockade of the metabolic syndrome in the V1bR-KO mice suggests additional mechanisms of protection beyond the upregulation of KHK. For example, V1b activation can regulate cortisol or glucagon, and it may also regulate vasopressin production itself (32). We also observed lower copeptin concentrations in our V1bR-KO mice, although concentrations were still 5-fold greater than baseline. Ideally, tissue-specific–KO mice of V1bR should facilitate the identification of the relative roles of cortisol and glucagon in driving this response.

In conclusion, sugar drives metabolic syndrome in part by the activation of vasopressin. Vasopressin drives fat production likely as a mechanism for storing metabolic water. The potential roles of hydration and salt reduction in the treatment of obesity and metabolic syndrome should be considered.

## Methods

### Animals.

KHK-A/C–KO (B6;129-Khk^tm2Dtb^) and KHK-A–KO (B6;129-Khk^tm2.1Dtb^) mice were originally developed by David Bonthorn (Leeds University, West Yorkshire, United Kingdom) (46) and were bred and maintained at the University of Colorado with pure C57/Bl6 for over 7 generations to ensure the mice were on the B6 genetic background. Mice with LoxP sequences flanking exons 3 and 4 of the *Khk* gene (*KHK^fl/fl^*) were generated by the Genomic Core at the University of Colorado Cancer Center. Liver-specific KHK-A/C–KO mice were obtained by crossing *KHK^fl/fl^* mice with liver-specific Cre-recombinase expressing mice obtained from Jackson Labs (*Cre-Alb* 003574). V1aR-KO (B6;129P2-*Avpr1a^tm1Dgen^*/J; 005776) and V1bR-KO (B6;129X1-*Avpr1b^tm1Wsy^*/J; 006160) mice were obtained from Jackson Labs. All experimental mice were maintained in temperature-controlled, humidity-controlled, specific pathogen–free conditions on a 14-hour dark/10-hour light cycle and at 25^o^C, and mice were allowed ad libitum access to normal laboratory chow (Harlan Teklad, 2920X). Water and food consumption were monitored daily and body weight recorded weekly for 30 weeks. Caloric intake was calculated as the sum of chow intake (3.1 cal/g) and water intake (accounting that fructose and glucose provide 4 cal/g). In all studies, 7- to 10-week-old male mice (*n* = 3–7) were employed. Food consumption was monitored daily and body weight recorded. All animals in the study were phenotypically normal and generally healthy during the study.

For 24-hour urinary collection, mice were acclimated to mouse metabolic cages for periods of 1–2 hours 1 week before collection. At the time of collection, animals were placed in metabolic cages and provided with chow and water (regular or with fructose) and the urinary cup was layered with a damp cloth to reduce potential urine evaporation. Urine was collected from cups every 6 hours to a total of 24 hours. Urinary fructose levels were determined biochemically following manufacturer’s instructions (BioAssay Systems, EFRU-100) and normalize to urinary creatinine.

We measured body composition, fat mass, and fat-free mass using quantitative MR (EchoMRI).

### Cell lines.

HepG2 cells were obtained from the ATCC (catalog HB-8065) and maintained in medium as recommended by the supplier. For experiments, cells were grown to 75% confluency before exposure to fructose (10 mM), AVP (250 nM), or in combination for 5 days. Medium was replaced twice daily and fresh fructose/AVP added in every change. Passages from 10 to 20 were employed for the experiments.

### Biochemical analysis.

Blood was collected in microtainer tubes (BD Biosciences) from cardiac puncture of mice under isoflurane, and serum was obtained after centrifugation at 13,148*g* for 2 minutes at room temperature. Serum parameters was performed biochemically following manufacturer’s instruction (uric acid: BioAssay Systems, DIUA-250; FGF21: R&D, MF2100; AST: BioAssay Systems, EASTR-100; ALT: BioAssay Systems, EALT-100; insulin: Crystal Chem, 90080; leptin: R&D, MOB00; FGF21: R&D, MF2100). Determination of parameters in tissue was performed in freeze-clamped tissues and measured biochemically following manufacturer’s protocol (triglycerides [liver]: BioAssay Systems, ETGA-200; uric acid: BioAssay Systems, DIUA-250).

### Histopathology.

Formalin-fixed paraffin-embedded liver, epididymal, and subscapular adipose sections were stained with H&E. Histological examination was performed through an entire cross-section of liver from each mouse. Images were captured on an Olympus BX51 microscope equipped with a 4-megapixel MacroFire Digital Camera (Optronics) using the PictureFrame Application 2.3 (Optronics). Composite images were assembled with the use of Adobe Photoshop. All images in each composite were handled identically.

### Western blot.

Protein lysates were prepared from mouse tissue employing MAP Kinase lysis buffer as previously described (47). Protein content was determined by the BCA protein assay (Pierce). Total protein (50 μg) was separated by SDS-PAGE (10% w/v) and transferred to PVDF membranes (BioRad). Membranes were first blocked for 1 hour at 25°C in 4% (w/v) instant milk dissolved in 0.1% Tween-20 Tris-Buffered Saline (TTBS); incubated with primary rabbit or mouse-raised antibodies (1:1000 dilution in TTBS) KHK (Sigma, HPA007040; RRID: AB_1079185), FAS (Cell Signaling, 3180; RRID: AB_2100796), ACC (Cell Signaling, 3676; RRID: AB_2219397), V1bR (BIOSS; bs-11800R), and actin (Cell Signaling, 4968; RRID: 2313904); and visualized using an anti-rabbit (7074; RRID: AB_2099233) or anti-mouse IgG (7076; RRID: AB_330924) horseradish peroxidase–conjugated secondary antibody (1:2000, Cell Signaling) using the HRP Immun-Star Detection Kit (Bio-Rad). Chemiluminescence was recorded with an Image Station 440CF and results analyzed with the 1D Image Software (Kodak Digital Science). See complete unedited blots in the supplemental material.

### Determination of KHK activity.

KHK activity on HepG2 cells was determined as previously described (48), with modifications. Briefly, HepG2 cells were incubated with vehicle control, AVP, or fructose for 5 days, as described in the text. After the incubation period, cells were lysed with a nondenaturing buffer containing 20 mM Tris-HCl, pH 7.5, 150 mM KCl, 1 mM EDTA, and 1 mM DTT, and centrifuged for 10 minutes at 13,148*g* at 4^o^C. The protein content of the supernatant fraction was quantified with the BCA Protein Assay Kit (Pierce), and KHK activity was measured on 50 μg lysate protein after addition of a buffer to 10 mM fructose in 50 mM imidazole, 1 M potassium acetate, pH 5.2, and 1 mM ATP. ATP was measured both before and after 2 hours incubation at 37°C using the ATP Determination Kit (BioVision, K354-100) as per manufacturer’s instructions. KHK activity was calculated as the ratio between ATP levels at 2 hours versus baseline for each sample at 0 time.

### Stably silencing of V1bR in HepG2 cells.

V1b deletion in HepG2 cells was performed employing lentiviral particles containing either shRNA sequences specific for human V1bR (Santa Cruz Biotechnologies, sc-40277-v) or scramble — noncodifying — shRNA control (sc-108080). Clonal selection was performed with puromycin (10 μg/mL) and validation of stably silenced clones was performed by Western blot at baseline and after 5 days of fructose (10 mM) exposure.

### Real-time PCR.

Cytosolic RNA was isolated from mouse tissues using the RNeasy Kit (Qiagen). Extraction of hypothalamic mRNA from mouse was performed as previously described (24). Before real-time PCR (RT-PCR), RNA was converted to cDNA using the iScript Reverse Transcriptase Kit (Bio-Rad) as described by the manufacturer. RT-PCR primers specific to *avpr1b* were obtained from Sigma (KICqStart, M_Avpr1b_1). RT-PCR was performed using 70 nM primers and the SYBR Green JumpStart *Taq* ReadyMix QPCR Kit (Sigma) on a Bio-Rad I-Cycler. RT-PCR runs were analyzed by agarose gel electrophoresis and melt curve to verify that the correct amplicon was produced. 18s RNA (KICqStart, M_Rn18s_1) was used as an internal control, and the amount of RNA was calculated by the comparative *C*_T_ method as recommended by the manufacturer.

### Insulin tolerance tests.

Insulin sensitivity was determined by both oral glucose and insulin tolerance tests as previously described (39).

### Statistics.

All numerical data are presented as mean ± SEM. Independent replicates for each data point (*n*) are identified in figure legends. Data graphics and statistical analysis were performed using Prism 5 (GraphPad). Data without indications were analyzed by 1-way ANOVA with Tukey post hoc test. A *P* value of less than 0.05 was regarded as statistically significant. Animals were randomly allocated in each group using Research Randomizer (https://www.randomizer.org). Power calculations for the number of animals assigned to each group were based on our previous publications and designed to observe a greater than 15% difference in body weight difference between groups. In general, an *n* of 6 mice per group was used. No animals were excluded from the study, and, whenever possible, experiments were done in a blinded fashion. For example, for data analysis, except for Western blot, single samples (serum, homogenates, etc.) were first codified and decoded after determination. Similarly, histological records and scoring were done in a blinded fashion.

### Study approval.

All animal experiments were conducted with adherence to the NIH *Guide for the Care and Use of Laboratory Animals* (National Academies Press, 2011). The animal protocol was approved by the Institutional Animal Care and Use Committee of the University of Colorado (Aurora).

## Author contributions

MAL and RJJ designed the research. MAL, AAH, DJO, TJJ, M. Kuwabara, RJJ, and MAL analyzed the data. CARJ, M. Kanbay, TI, PSM, PB, TN, LGSL, RJJ, and MAL provided key resources and expertise. AAH, TJJ, M. Kuwabara, CC, NL, GEG, and MAL performed the research. RJJ and MAL wrote the paper.

## Supplementary Material

Supplemental data

## Version 1 12/15/2020

In-Press Preview

## Version 2 01/11/2021

Electronic publication
