# Supplementary material for: Vasopressin mediates fructose-induced metabolic syndrome by activating the V1b receptor
Source: JCI Insight. 2021 Jan 11;6(1):e140848. doi: 10.1172/jci.insight.140848 (PMC7821599; doi:10.1172/jci.insight.140848)

**Supplemental Materials:**

**Supplemental Figure 1. Hydrogel therapy result in increased total water intake and vasopressin lowering in mice. A)** Comparative image of the aspect of regular chow and hydrogel chow pellets. **B)** Averaged daily total water intake in wild type mice receiving regular chow (normal water intake, NWI, black bars) or hydrogels (high water intake HWI, blue bars) for 7 days. **C)** Serum osmolality in NWI and HWI groups at day 7. **D)** Urinary osmolality in NWI and HWI groups at day 7. **E)** Serum copeptin levels in NWI and HWI groups at day 7.The data in B-E) were presented as the means ± SD and analyzed by two-tail ttest. $^{**}P < 0.01$. $n = 6$ mice per group. This figure is related to Figures 3-4 and Supplemental Tables 3-4.

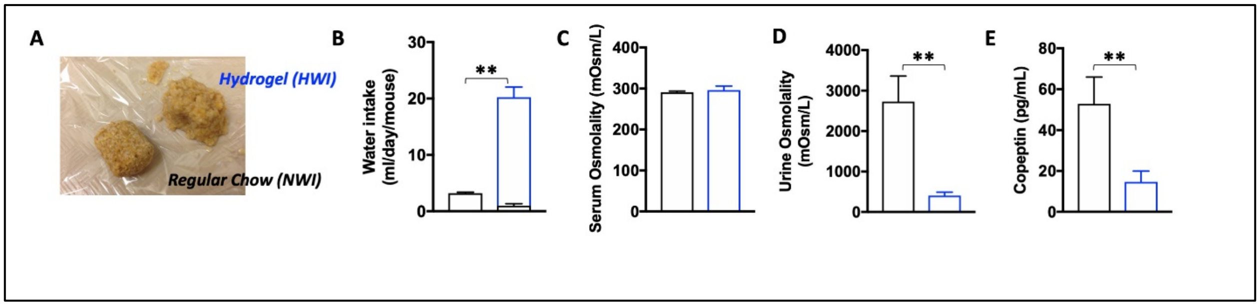

**Supplemental Table 1: General Parameters in wild type (WT) and fructokinase knockout (KHK-A and KHK-A/C KO) mice exposed to water control or fructose (Related to Figure 2).** * *P*<0.05 and ** *P < 0.01* versus respective water controls. One way ANOVA Tukey post hoc analysis, *n* = 6 mice per group.

| | **WT Water (*n* = 6)** | **WT fructose 15 % (*n* = 6)** | **KHK-A KO water (*n* = 6)** | **KHK-A KO fructose 15 % (*n* = 6)** | **KHK-A/C KO Water (*n* = 6)** | **KHK-A/C KO fructose 30% (*n* = 6)** | **WT fructose 15 % vs KHK-A/C KO fructose 30 % ANOVA** |
|---|---|---|---|---|---|---|---|
| **Body Weight and composition** | | | | | | | |
| Body Weight; 0W (g) | 23.7 ± 0.4 | 23.8 ± 0.2 | 22.7 ± 0.3 | 22.6 ± 0.3 | 23.4 ± 0.6 | 23.5 ± 0.7 | *NS* |
| Body Weight; 30W (g) | 35.7 ± 3.2 | **45.7 ± 3.6**** | 36.2 ± 2.7 | **47.6 ± 2.6**** | 33.6 ± 3.6 | 35.1 ± 1.6 | *P* < 0.01 |
| ΔBody Weight; 30W (g) | 12.0 ± 2.6 | **21.9 ± 2.6**** | 13.5 ± 2.7 | 25.0 ± 2.1** | 10.2 ± 2.6 | 11.6 ± 0.9 | *P* < 0.01 |
| Average Food Intake (g/day) | 3.31 ± 0.2 | **2.74 ± 0.3*** | 3.38 ± 0.2 | **2.82 ± 0.2*** | 3.05 ± 0.3 | **2.46 ± 0.2**** | *NS* |
| Water Intake (ml/day) | 3.1 ± 0.2 | **6.7 ± 0.7**** | 3.2 ± 0.3 | **6.4 ± 0.6**** | 3.1 ± 0.3 | 3.4 ± 0.5 | *P* < 0.01 |
| Fructose intake (g/day) | 0 | **1.01 ± 0.11**** | **0** | **0.96 ± 0.09**** | 0 | **1.02 ± 0.105**** | *NS* |
| Cumulative Caloric Intake 30W | 2363 ± 86 | **2750 ± 88**** | 2413 ± 102 | **2719 ± 75**** | 2177 ± 38 | **2641 ± 105**** | *NS* |
| Cumulative Caloric Intake/BW 30W | 66.2 ± 9 | 60.2 ± 23 | 66.6 ± 25 | 57.1 ± 16 | 64.8 ± 22 | 75.2 ± 17 | *NS* |
| Cumulative Calories from Fructose; 30W | 0 | **844 ± 36**** | 0 | **806 ± 23**** | 0 | **856 ± 25**** | *NS* |
| Cumulative Calories from Fructose/BW 30W | 0 | **18.5 ± 8*** | 0 | **16.9 ± 6*** | 0 | **24.3 ± 7**** | *NS* |
| Hypothalamic *avp* mRNA (A.U.) | 1.16 ± 0.3 | **3.83 ± 0.8**** | 0.97 ± 0.3 | **4.42 ± 1.04**** | 1.36 ± 0.5 | 1.25 ± 0.8 | *P* < 0.01 |
| Pituitary AVP (ng/pituitary) | 46 ± 21 | **193 ± 57**** | 58 ± 17 | **237 ± 52**** | 61 ± 18 | 82 ± 32 | *P* < 0.01 |
| Serum copeptin | 64 ± 22 | **192 ± 14**** | 86 ± 13 | **197 ± 19**** | 69 ± 10 | 93 ± 27 | *P* < 0.01 |

| | | | | | | | |
|---|---|---|---|---|---|---|---|
| (pg/ml) | | | | | | | |
| Serum Osmolality (mOsm/L) | 301 ± 5 | 298 ± 5 | 296 ± 5 | 301 ± 3 | 297 ± 4 | 296 ± 3 | *NS* |
| Urine Osmolality (mOsm/L) | 2765 ± 310 | **415 ± 155**** | 2283 ± 355 | **610 ± 205**** | 2585 ± 255 | 2675 ± 355 | *P* < 0.01 |
| Urine Fructose (nmol/mg Cre) | 33.1 ± 12.2 | 41.1 ± 12.6 | 21.6 ± 11.3 | 34.6 ± 16 | 127.7 ± 41.3 | **448 ± 161**** | *P* < 0.01 |
| Liver Weight; 30 W (g) | 1.64 ± 0.2 | **2.52 ± 0.3**** | 1.55 ± 0.30 | **2.46 ± 0.3**** | 1.27 ± 0.12 | 1.46 ± 0.15 | *P* < 0.01 |
| Fat Mass (g) | 7.1 ± 2.3 | **20.8 ± 4.5**** | 8.2 ± 3.3 | **22.6 ± 5.0**** | 7.8 ± 2.8 | 8.1 ± 2.5 | *P* < 0.01 |
| Fat Mass (% BW) | 19.8 ± 3.6 | **45.5 ± 6.3**** | 22.6 ± 4.8 | **47.4 ± 8.5**** | 23.2 ± 4.9 | 23.1 ± 4.3 | *P* < 0.01 |
| **Biochemical Blood Analysis** | | | | | | | |
| AST (IU/L) | 41 ± 7 | **106 ± 15**** | 45 ± 17 | **114 ± 14**** | 33 ± 9 | 39 ± 3 | *P* < 0.01 |
| ALT (IU/L) | 27 ± 8 | **73 ± 11**** | 22 ± 5 | **89 ± 10**** | 21 ± 7 | 25 ± 7 | *P* < 0.01 |
| Serum Triglycerides (mg/dl) | 53.6 ± 7.2 | 64.2 ± 8.6 | 43.7 ± 6.7 | **64.6 ± 9.5**** | 36.6 ± 7.2 | 34.3 ± 5.7 | *P* < 0.01 |
| Fasting Serum Glucose (mg/dl) | 94 ± 5 | 104 ± 5 | 91 ± 8 | **106 ± 3**** | 94 ± 6 | 100 ± 8 | *NS* |
| Fasting Insulin (ng/ml) | 0.7 ± 0.2 | **4.1 ± 1.1**** | 0.6 ± 0.2 | **5.6 ± 1.4**** | 0.6 ± 0.2 | 0.7 ± 0.2 | *P* < 0.01 |
| OGTT (AUC/10) | 336 ± 42 | **556 ± 51**** | 277 ± 25 | **570 ± 31**** | 313 ± 33 | 341 ± 26 | *P* < 0.01 |
| Fasting Leptin (ng/ml) | 10.2 ± 3.6 | **41.6 ± 8.2**** | 8.8 ± 2.5 | **47.2 ± 10.2**** | 6.6 ± 3.8 | 11.2 ± 4.5 | *P* < 0.01 |
| FGF21 (pg/ml) | 283 ± 56 | **4245 ± 315**** | 410 ± 62 | **5155 ± 425**** | 210 ± 23 | **616 ± 145*** | *P* < 0.01 |
| **Liver Analysis** | | | | | | | |
| Triglycerides (mg/g) | 31 ± 15 | **123 ± 22**** | 41 ± 16 | **144 ± 26**** | 26 ± 8 | 31 ± 10 | *P* < 0.01 |

**Supplemental Table 2: General Parameters in floxed fructokinase (*KHK*$^{Fl/FL}$) and liver-specific fructokinase knockout (*KHK*$^{Fl/FL}$*XCreAlb*) mice exposed to water control or fructose (Related to Figure 2).** * *P*<0.05 and ** *P < 0.01* versus respective water controls. One way ANOVA Tukey post hoc analysis, *n* = 6 mice per group.

| | ***KHK*$^{Fl/FL}$ Water (*n* = 6)** | ***KHK*$^{Fl/FL}$ fructose 15 % (*n* = 6)** | ***KHK*$^{Fl/FL}$*XCreAlb* Water (*n* = 6)** | ***KHK*$^{Fl/FL}$*XCreAlb* fructose 15 % (*n* = 6)** | ***KHK*$^{Fl/FL}$ fructose 15 % vs *KHK*$^{Fl/FL}$*XCreAlb* fructose 15 % ANOVA** |
|---|---|---|---|---|---|
| **Body Weight and composition** | | | | | |
| Body Weight; 0W (g) | 24.2 ± 0.6 | 23.6 ± 0.5 | 23.7 ± 0.6 | 24.1 ± 0.6 | *NS* |
| Body Weight; 30W (g) | 34.8 ± 2.9 | **43.9 ± 4.6**** | 32.9 ± 1.6 | 34.1 ± 1.9 | *P* < 0.01 |
| ΔBody Weight; 30W (g) | 10.6 ± 1.3 | **20.3 ± 3.0**** | 9.2 ± 1.1 | 10.0 ± 0.9 | *P* < 0.01 |
| Average Food Intake (g/day) | 3.35 ± 0.2 | **2.87 ± 0.2*** | 3.15 ± 0.2 | **2.34 ± 0.3**** | *NS* |
| Water Intake (ml/day) | 3.2 ± 0.2 | **7.1 ± 0.4**** | 3.1 ± 0.4 | **7.2 ± 0.4**** | *NS* |
| Fructose intake (g/day) | 0 | **1.06 ± 0.10**** | 0 | **1.028 ± 0.12**** | *NS* |
| Cumulative Caloric Intake 30W | 2427 ± 185 | **2865 ± 190**** | 2213 ± 156 | **2620 ± 105**** | *P* < 0.01 |
| Cumulative Caloric Intake/BW 30W | 69.7 ± 19 | 65.2 ± 13 | 67.2 ± 15 | 76.8 ± 14 | *NS* |
| Cumulative Calories from Fructose; 30W | 0 | **894 ± 40**** | 0 | **907 ± 31**** | *NS* |
| Cumulative Calories from Fructose/BW 30W | 0 | **20.4 ± 9*** | 0 | **26.5 ± 11**** | *NS* |
| Hypothalamic *avp* mRNA (A.U.) | 1.03 ± 0.2 | **4.71 ± 0.6**** | 0.92 ± 0.4 | 1.56 ± 0.3 | *P* < 0.01 |
| Pituitary AVP (ng/pituitary) | 35 ± 13 | **171 ± 36**** | 51 ± 23 | **116 ± 22**** | *P* < 0.01 |
| Serum copeptin (pg/ml) | 55 ± 16 | **189 ± 20**** | 44 ± 18 | **133 ± 22**** | *P* < 0.01 |
| Serum Osmolality (mOsm/L) | 302 ± 4 | 304 ± 4 | 299 ± 6 | 298 ± 5 | *NS* |
| Urine Osmolality (mOsm/L) | 2556 ± 273 | **490 ± 35**** | 2267 ± 145 | **475 ± 35**** | *NS* |
| Urine Fructose (nmol/mg Cre) | 22.1 ± 8.6 | 22.3 ± 9.1 | 65.8 ± 16.2 | **416 ± 135**** | *P* < 0.01 |

| | | | | | |
|---|---|---|---|---|---|
| Liver Weight; 30 W (g) | 1.43 ± 0.3 | **2.65 ± 0.3**** | 1.36 ± 0.14 | 1.41 ± 0.2 | *P* < 0.01 |
| Fat Mass (g) | 8.8 ± 2.5 | **21.4 ± 3.6**** | 8.3 ± 1.4 | 9.3 ± 2.8 | *P* < 0.01 |
| Fat Mass (% BW) | 25.2 ± 1.6 | **48.7 ± 2.4**** | 25.2 ± 2.9 | 27.2 ± 2.7 | *P* < 0.01 |
| **Biochemical Blood Analysis** | | | | | |
| AST (IU/L) | 36 ± 10 | **121 ± 8**** | 39 ± 4 | 34 ± 7 | *P* < 0.01 |
| ALT (IU/L) | 25 ± 10 | **89 ± 12**** | 26 ± 5 | 25 ± 5 | *P* < 0.01 |
| Serum Triglycerides (mg/dl) | 46.2 ± 6.6 | **67.1 ± 5.4**** | 34.3 ± 5.2 | 29.6 ± 6.2 | *P* < 0.01 |
| Fasting Serum Glucose (mg/dl) | 100 ± 6 | 103 ± 4 | 97 ± 5 | 95 ± 7 | *NS* |
| Fasting Insulin (ng/ml) | 0.6 ± 0.2 | **4.2 ± 1.2**** | 0.6 ± 0.2 | 0.6 ± 0.2 | *P* < 0.01 |
| OGTT (AUC/10) | 371 ± 36 | **512 ± 35**** | 341 ± 40 | 356 ± 29 | *P* < 0.01 |
| Fasting Leptin (ng/ml) | 9.4 ± 3.6 | **51.1 ± 10.1**** | 10.3 ± 5.1 | 9.6 ± 3.3 | *P* < 0.01 |
| FGF21 (pg/ml) | 313 ± 35 | **5112 ± 263**** | 343 ± 35 | **916 ± 233**** | *P* < 0.01 |
| **Liver Analysis** | | | | | |
| Triglycerides (mg/g) | 42 ± 13 | **151 ± 16**** | 33 ± 10 | 37 ± 8 | *P* < 0.01 |

**Supplemental Table 3: General Parameters in wild type mice on regular water or high fructose corn syrup (HFCS) under normal or high water intake (HWI) conditions via hydrogels (Related to Figure 3).** * $P<0.05$ and ** $P < 0.01$ versus respective water controls. One way ANOVA Tukey post hoc analysis, *n* = 6 mice per group.

| | ***WT* Water (*n* = 6)** | **WT HFCS 10 % (*n* = 6)** | ***WT* HWI (*n* = 6)** | ***WT* HFCS-HWI (*n* = 6)** | ***WT* HFCS 10 % vs *WT HFCS-HWI 10 %* ANOVA** |
|---|---|---|---|---|---|
| **Body Weight and composition** | | | | | |
| Body Weight; 0W (g) | 22.3 ± 1.4 | 22.0 ± 2.6 | 23.1 ± 0.6 | 23.1 ± 0.8 | *NS* |
| Body Weight; 30W (g) | 35.6 ± 3.3 | **48.4 ± 2.6**** | 36.0 ± 3.1 | 39.6 ± 1.6 | *P* < 0.01 |
| ΔBody Weight; 30W (g) | 13.3 ± 2.6 | **26.4 ± 2.2**** | 12.9 ± 2.1 | 13.5 ± 1.2 | *P* < 0.01 |
| Average Food Intake (g/day) | 3.11 ± 0.2 | 2.76 ± 0.2 | 3.45 ± 0.4 | **2.33 ± 0.3**** | *NS* |
| Water Intake (ml/day) | 3.1 ± 0.2 | **15.8 ± 2.6**** | 29.9 ± 5.6 | **25.7 ± 5.1*** | *P* < 0.01 |
| Fructose intake (g/day) | 0 | **0.94 ± 0.21**** | 0 | **0.98 ± 0.16**** | *NS* |
| Cumulative Caloric Intake 30W | 2259 ± 210 | **3245 ± 335**** | 2390 ± 216 | **2993 ± 105**** | *NS* |
| Cumulative Caloric Intake/BW 30W | 63.4 ± 16 | 67.0 ± 12 | 71.5 ± 8 | 75.5 ± 12 | *NS* |
| Cumulative Calories from Fructose; 30W | 0 | **797 ± 35**** | 0 | **825 ± 52**** | *NS* |
| Cumulative Calories from Fructose/BW 30W | 0 | **16.4 ± 6*** | 0 | **20.8 ± 10**** | *NS* |
| Hypothalamic *avp* mRNA (A.U.) | 0.99 ± 0.3 | **3.73 ± 0.3**** | 0.56 ± 0.3 | 0.73 ± 0.2 | *P* < 0.01 |
| Pituitary AVP (ng/pituitary) | 31 ± 8 | **136 ± 13**** | 24 ± 6 | 20 ± 6 | *P* < 0.01 |
| Serum copeptin (pg/ml) | 21 ± 10 | **390 ± 66**** | 10 ± 6 | 43 ± 18 | *P* < 0.01 |
| Serum Osmolality (mOsm/L) | 297 ± 4 | 303 ± 6 | 297 ± 5 | 295 ± 5 | *NS* |
| Urine Osmolality (mOsm/L) | 2290 ± 323 | **412 ± 40**** | 356 ± 12 | 388 ± 21 | *P* < 0.01 |
| Urine Fructose (nmol/mg Cre) | 21.6 ± 2.1 | 24.3 ± 6.7 | 20.2 ± 1.9 | **35.7 ± 5**** | *P* < 0.01 |

| | | | | | |
|---|---|---|---|---|---|
| Liver Weight; 30 W (g) | 1.31 ± 0.3 | **2.46 ± 0.32**** | 1.41 ± 0.2 | 1.67 ± 0.2 | *P* < 0.01 |
| Fat Mass (g) | 7.7 ± 1.5 | **22.1 ± 2.3**** | 5.7 ± 1.9 | **9.3 ± 2.1*** | *P* < 0.01 |
| Fat Mass (% BW) | 21.6 ± 6.4 | **46.1 ± 6.9**** | 16.5 ± 5.3 | 24.2 ± 6.5 | *P* < 0.01 |
| **Biochemical Blood Analysis** | | | | | |
| AST (IU/L) | 32 ± 8 | **116 ± 13**** | 26 ± 6 | 27 ± 5 | *P* < 0.01 |
| ALT (IU/L) | 43 ± 7 | **121 ± 14**** | 43 ± 11 | 43 ± 8 | *P* < 0.01 |
| Serum Triglycerides (mg/dl) | 38.5 ± 4.5 | **49.6 ± 6.1**** | 27.7 ± 3.3 | 31.3 ± 4.2 | *P* < 0.01 |
| Fasting Serum Glucose (mg/dl) | 96 ± 4 | 101 ± 5 | 99 ± 5 | 96 ± 3 | *NS* |
| Fasting Insulin (ng/ml) | 0.5 ± 0.2 | **3.4 ± 0.6**** | 0.4 ± 0.2 | 0.6 ± 0.2 | *P* < 0.01 |
| OGTT (AUC/10) | 356 ± 24 | **568 ± 41**** | 336 ± 21 | 312 ± 49 | *P* < 0.01 |
| Fasting Leptin (ng/ml) | 15.5 ± 4.2 | **55.5 ± 12.5**** | 13.6 ± 3.2 | 20.1 ± 7.2 | *P* < 0.01 |
| FGF21 (pg/ml) | 466 ± 41 | **4785 ± 312**** | 355 ± 25 | **1085 ± 225**** | *P* < 0.01 |
| **Liver Analysis** | | | | | |
| Triglycerides (mg/g) | 56 ± 7 | **145 ± 22**** | 39 ± 13 | 43 ± 8 | *P* < 0.01 |

**Supplemental Table 4: General Parameters in wild type mice on regular water or high fructose corn syrup (HFCS) under normal or high water intake (HWI) conditions via hydrogels after week 15 (Related to Figure 4).** * *P*<0.05 and ** *P < 0.01* versus water control. One way ANOVA Tukey post hoc analysis, *n* = 6 mice per group.

| | ***WT* Water (*n* = 6)** | **WT HFCS 10 % (*n* = 6)** | ***WT* HFCS-HWI 10 % (*n* = 6)** | ***WT* HFCS 10 % vs *WT HFCS-HWI 10 %* ANOVA** |
|---|---|---|---|---|
| **Body Weight and composition** | | | | |
| Body Weight; 0W (g) | 23.4 ± 2.4 | 23.3 ± 1.6 | 23.4 ± 0.4 | *NS* |
| Body Weight; 30W (g) | 34.5 ± 3.8 | **51.1 ± 5.2**** | 39.0 ± 3.3 | *P* < 0.01 |
| ΔBody Weight; 30W (g) | 11.1 ± 2.7 | **27.8 ± 4.1**** | 15.6 ± 2.7 | *P* < 0.01 |

| | | | | |
|---|---|---|---|---|
| ΔBody Weight; 15-30W (g) | 5.1 ± 1.1 | **9.1 ± 2.5*** | **-2.1 ± 3.1**** | *P* < 0.01 |
| Average Food Intake (g/day) | 3.26 ± 0.2 | **2.81 ± 0.2*** | **2.64 ± 0.3**** | *NS* |
| Average Food Intake 15-30W (g/day) | 3.17 ± 0.2 | 2.93 ± 0.2 | **2.21 ± 0.3**** | *P* < 0.01 |
| Water Intake (ml/day) | 2.9 ± 0.2 | **16.4 ± 3.2**** | **21.6 ± 5.8**** | *NS* |
| Water Intake 15-30W (ml/day) | 3.2 ± 0.2 | **17.2 ± 2.7**** | **26.6 ± 4.6**** | *P* < 0.01 |
| Fructose intake (g/day) | 0 | **0.98 ± 0.14**** | **1.29 ± 0.21**** | *NS* |
| Fructose intake 15-30W (g/day) | 0 | **1.03 ± 0.12**** | **1.59 ± 0.19**** | *P* < 0.01 |
| Cumulative Caloric Intake 30W | 2217 ± 165 | **3318 ± 285**** | **3116 ± 313**** | *NS* |
| Cumulative Caloric Intake 15-30W | 1151 ± 76 | **1737 ± 167**** | **1619 ± 180**** | *NS* |
| Cumulative Caloric Intake/BW 30W | 64.2 ± 19 | 64.9 ± 16 | 79.8 ± 23 | *NS* |
| Cumulative Calories from Fructose; 30W | 0 | **823 ± 26**** | **1083 ± 67**** | *P* < 0.01 |
| Cumulative Calories from Fructose; 15-30W | 0 | **432 ± 31**** | **667 ± 42**** | *P* < 0.01 |
| Cumulative Calories from Fructose/BW 30W | 0 | **16.1 ± 5**** | **27.7 ± 11**** | *P* < 0.05 |
| Hypothalamic *avp* mRNA (A.U.) | 0.95 ± 0.4 | **4.21 ± 0.3**** | 1.13 ± 0.3 | *P* < 0.01 |
| Pituitary AVP (ng/pituitary) | 43 ± 12 | **132 ± 10**** | 27 ± 8 | *P* < 0.01 |
| Serum copeptin (pg/ml) | 31 ± 14 | **480 ± 120**** | **135 ± 26**** | *P* < 0.01 |
| Serum copeptin Δ15-30W (pg/ml) | 15 ± 6 | **117 ± 31**** | **-208 ± 21**** | *P* < 0.01 |
| Serum Osmolality (mOsm/L) | 302 ± 5 | 303 ± 5 | 299 ± 6 | *NS* |
| Urine Osmolality (mOsm/L) | 2478 ± 480 | **516 ± 81**** | **363 ± 41**** | *P* < 0.01 |
| Urine Fructose (nmol/mg Cre) | 13.3 ± 3.1 | 16.8 ± 8.1 | **32.8 ± 9.2**** | *P* < 0.01 |
| Liver Weight; 30 W (g) | 1.44 ± 0.3 | **2.61 ± 0.3**** | **1.97 ± 0.2*** | *P* < 0.01 |

| | | | | |
|---|---|---|---|---|
| Fat Mass (g) | 6.6 ± 1.8 | **21.2 ± 3.5**** | **11.5 ± 2.6*** | *P* < 0.01 |
| Fat Mass (% BW) | 19.1 ± 3.3 | **41.6 ± 8.2**** | **29.6 ± 5.2**** | *P* < 0.01 |
| **Biochemical Blood Analysis** | | | | |
| AST (IU/L) | 37 ± 6 | **128 ± 24**** | 52 ± 16 | *P* < 0.01 |
| ALT (IU/L) | 42 ± 5 | **136 ± 12**** | 42 ± 11 | *P* < 0.01 |
| Serum Triglycerides (mg/dl) | 46.2 ± 5.1 | **57.3 ± 4.8**** | 38.5 ± 6.6 | *P* < 0.01 |
| Fasting Serum Glucose (mg/dl) | 102 ± 4 | 98 ± 4 | 99 ± 4 | *NS* |
| Fasting Insulin (ng/ml) | 0.6 ± 0.3 | **3.6 ± 0.4**** | 0.5 ± 0.2 | *P* < 0.01 |
| OGTT (AUC/10) | 332 ± 32 | **531 ± 27**** | 387 ± 43 | *P* < 0.01 |
| Fasting Leptin (ng/ml) | 34.3 ± 6.3 | **67.2 ± 11.2**** | **44.3 ± 8.3*** | *P* < 0.01 |
| FGF21 (pg/ml) | 296 ± 110 | **4903 ± 225**** | **2356 ± 410**** | *P* < 0.01 |
| **Liver Analysis** | | | | |
| Triglycerides (mg/g) | 42 ± 11 | **139 ± `16**** | **74 ± 12**** | *P* < 0.01 |

**Supplemental Table 5: General Parameters in wild type (WT), vasopressin 1a receptor KO (V1aR) and vasopressin 1b receptor KO (V1bR) mice exposed to water control or fructose (Related to Figure 5).** * *P*<0.05 and ** *P < 0.01* versus respective water controls. One way ANOVA Tukey post hoc analysis, *n* = 6 mice per group.

| | **WT Water (*n* = 6)** | **WT fructose 15 % (*n* = 6)** | **V1aR KO water (*n* = 6)** | **V1aR KO fructose 15 % (*n* = 6)** | **V1bR KO Water (*n* = 6)** | **V1bR KO fructose 15 % (*n* = 6)** | **WT 15 % vs V1bR 15% fructose 15 % ANOVA** |
|---|---|---|---|---|---|---|---|
| **Body Weight and composition** | | | | | | | |
| Body Weight; 0W (g) | 22.8 ± 0.7 | 23.7 ± 0.5 | 23.4 ± 1.1 | 24.1 ± 0.7 | 23.1 ± 0.6 | 22.6 ± 1.7 | *NS* |
| Body Weight; 30W (g) | 36.4 ± 2.8 | **49.9 ± 5.7**** | 38.1 ± 4.1 | **56.3 ± 3.5**** | 34.7 ± 2.2 | 35.8 ± 3.1 | *P* < 0.01 |
| ΔBody Weight; 30W (g) | 13.6 ± 2.1 | **26.2 ± 3.8**** | 14.7 ± 2.9 | **32.2 ± 2.6**** | 11.6 ± 1.4 | 13.2 ± 2.7 | *P* < 0.01 |
| Average Food Intake (g/day) | 3.23 ± 0.2 | **2.77 ± 0.3*** | 3.35 ± 0.2 | 3.11 ± 0.3 | 2.95 ± 0.3 | **2.21 ± 0.2**** | *P* < 0.01 |
| Water Intake (ml/day) | 3.2 ± 0.2 | **7.1 ± 1.1**** | 3.4 ± 0.3 | **7.5 ± 0.5**** | 3.3 ± 0.3 | **8.4 ± 0.7**** | *P* < 0.01 |
| Fructose intake (g/day) | 0 | **1.09 ± 0.16**** | **0** | **1.15 ± 0.12**** | 0 | **1.26 ± 0.21**** | *NS* |
| Cumulative Caloric Intake 30W | 2283 ± 163 | **2910 ± 155**** | 2356 ± 223 | **3268 ± 135**** | 2090± 116 | **2583 ± 190**** | *P* < 0.05 |
| Cumulative Caloric Intake/BW 30W | 62.7 ± 13 | 58.3 ± 11 | 61.8 ± 11 | 58.0 ± 16 | 60.2 ± 14 | 72.1 ± 15 | *NS* |
| Cumulative Calories from Fructose; 30W | 0 | **864 ± 45**** | 0 | **893 ± 41**** | 0 | **916 ± 23**** | *NS* |
| Cumulative Calories from Fructose/BW 30W | 0 | **17.3 ± 6*** | 0 | **15.8 ± 5*** | 0 | **25.6 ± 10**** | *NS* |
| Hypothalamic *avp* mRNA (A.U.) | 0.97 ± 0.3 | **3.76 ± 0.5**** | 1.05 ± 0.3 | **3.67 ± 0.7**** | 0.93 ± 0.2 | 1.66 ± 0.4 | *P* < 0.01 |
| Pituitary AVP (ng/pituitary) | 41 ± 11 | **157 ± 22**** | 51 ± 9 | **212 ± 32**** | 36 ± 13 | **92 ± 21**** | *P* < 0.01 |

| | | | | | | | |
|---|---|---|---|---|---|---|---|
| Serum copeptin (pg/ml) | 52 ± 16 | **394 ± 23**** | 59 ± 11 | **448 ± 19**** | 51 ± 8 | **240 ± 13**** | *P* < 0.01 |
| Serum Osmolality (mOsm/L) | 303 ± 3 | 301 ± 3 | 304 ± 2 | 303 ± 1 | 297 ± 3 | 301 ± 3 | *NS* |
| Urine Osmolality (mOsm/L) | 2510 ± 235 | **436 ± 46**** | 2110 ± 180 | **485 ± 115**** | 2390 ± 142 | **410 ± 45**** | *NS* |
| Urine Fructose (nmol/mg Cre) | 17.7 ± 9.1 | 23.3 ± 6.1 | 23.4 ± 12.2 | 27.3 ± 4.4 | 31.2 ± 8.8 | **56.7 ± 14**** | *P* < 0.01 |
| Liver Weight; 30 W (g) | 1.48 ± 0.2 | **2.59 ± 0.3**** | 1.67 ± 0.2 | **2.95 ± 0.2**** | 1.41 ± 0.2 | 1.55 ± 0.2 | *P* < 0.01 |
| Fat Mass (g) | 8.6 ± 1.7 | **16.9 ± 1.6**** | 11.2 ± 2.6 | **20.1 ± 2.4**** | 7.4 ± 2.3 | 10.3 ± 2.2 | *P* < 0.01 |
| Fat Mass (% BW) | 20.2 ± 5.1 | **36.6 ± 3.0**** | 21.8 ± 2.9 | **42.6 ± 2.5**** | 21.4 ± 5.1 | 26.8 ± 3.9 | *P* < 0.01 |
| **Biochemical Blood Analysis** | | | | | | | |
| AST (IU/L) | 36 ± 5 | **114 ± 28**** | 44 ± 9 | **133 ± 10**** | 26 ± 5 | 31 ± 3 | *P* < 0.01 |
| ALT (IU/L) | 24 ± 6 | **106 ± 11**** | 29 ± 5 | **127 ± 8**** | 26 ± 6 | 34 ± 5 | *P* < 0.01 |
| Serum Triglycerides (mg/dl) | 47.4 ± 6.1 | **66.6 ± 6.6**** | 53.4 ± 5.4 | **71.2 ± 4.8**** | 41.0 ± 3.5 | 37.3 ± 4.6 | *P* < 0.01 |
| Fasting Serum Glucose (mg/dl) | 100 ± 4 | 105 ± 6 | 97 ± 5 | 104 ± 3 | 102 ± 5 | 100 ± 6 | *NS* |
| Fasting Insulin (ng/ml) | 0.6 ± 0.2 | **2.8 ± 0.6**** | 0.6 ± 0.2 | **3.8 ± 0.7**** | 0.5 ± 0.2 | 0.5 ± 0.2 | *P* < 0.01 |
| OGTT (AUC/10) | 421 ± 23 | **612 ± 40**** | 381 ± 33 | **667 ± 55**** | 397 ± 45 | 328 ± 21 | *P* < 0.01 |
| Fasting Leptin (ng/ml) | 7.9 ± 2.1 | **60.1 ± 8.2**** | 12.3 ± 2.2 | **67.8 ± 10.0**** | 9.9 ± 2.7 | 18.6 ± 4.1 | *P* < 0.01 |
| FGF21 (pg/ml) | 401 ± 61 | **4661 ± 223**** | 490 ± 33 | **5712 ± 661**** | 275 ± 31 | 788 ± 156 | *P* < 0.01 |
| **Liver Analysis** | | | | | | | |
| Triglycerides (mg/g) | 43 ± 12 | **128 ± 19**** | 57 ± 8 | **192 ± 20**** | 31 ± 6 | **61 ± 19*** | *P* < 0.01 |

Full unedited images:
Full unedited images for Figure 2F
**KHK**

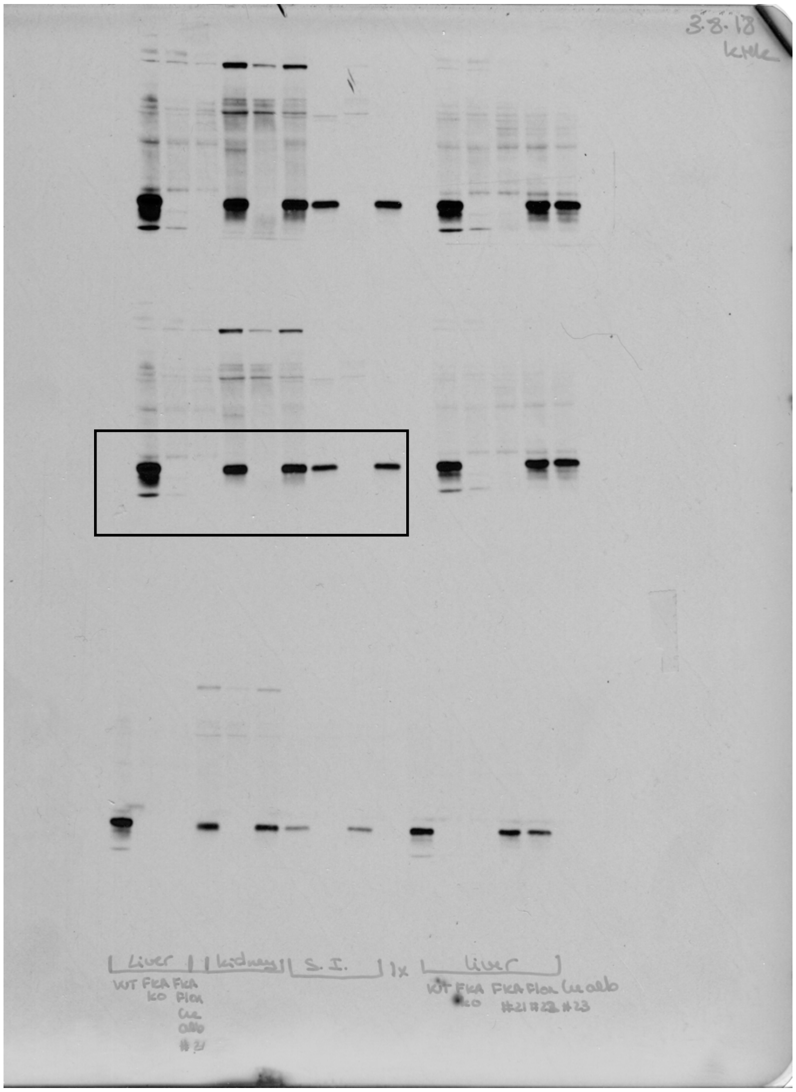

**GAPDH (stripped and reblotted from KHK)**

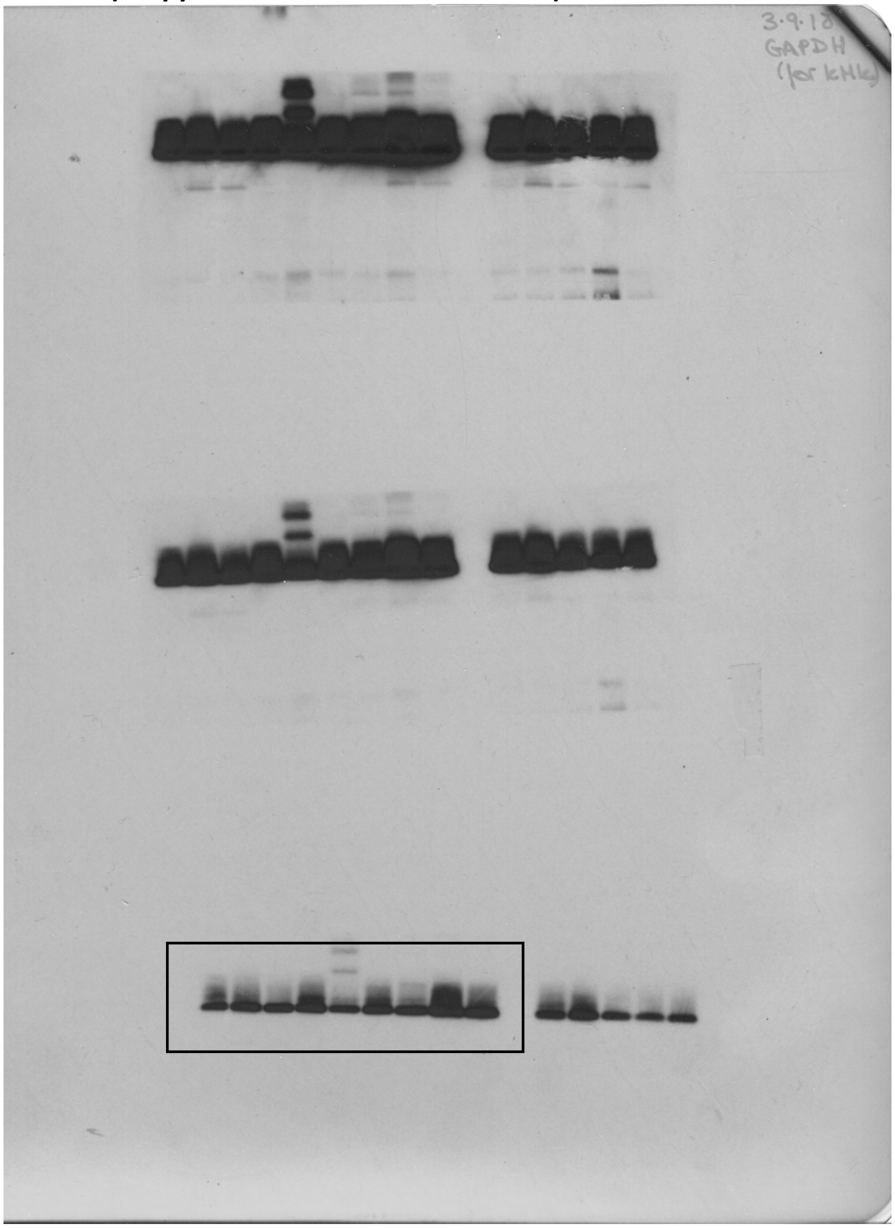

Full unedited images for figure 6C

**KHK (left)** **V1b (right)**

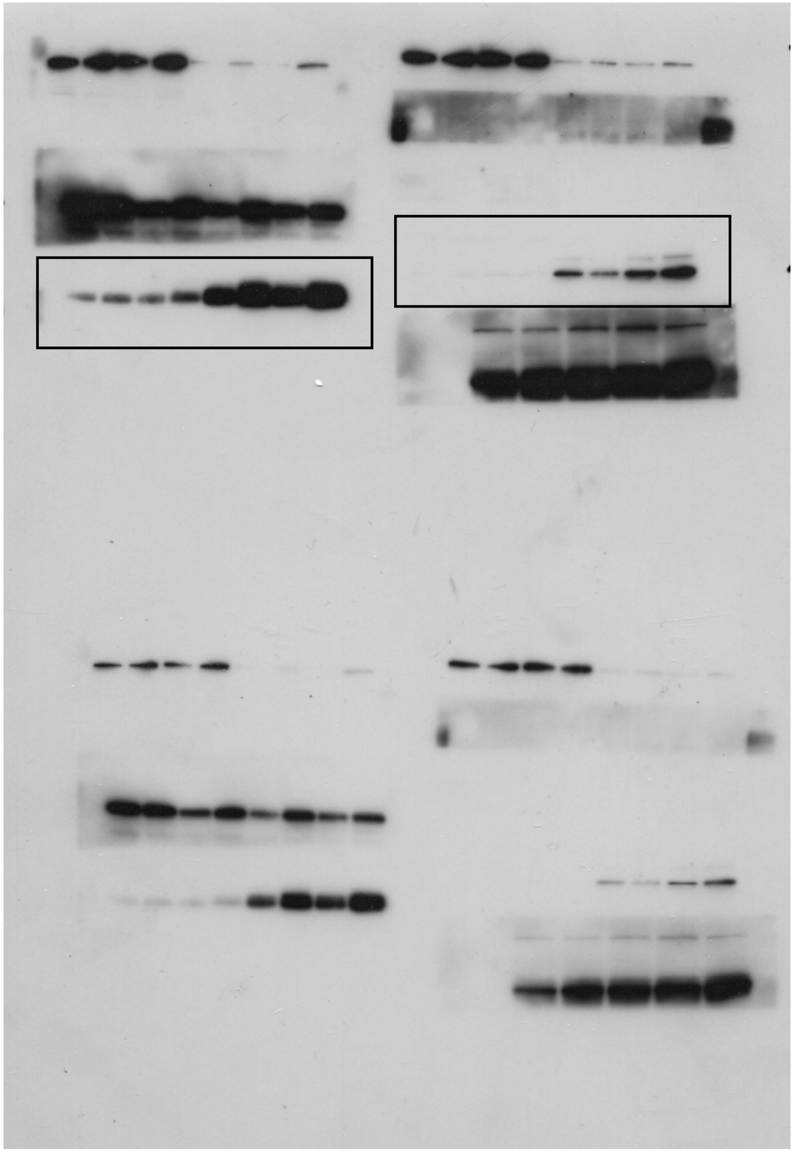

**Actin (stripped and reblotted from KHK and V1b)**

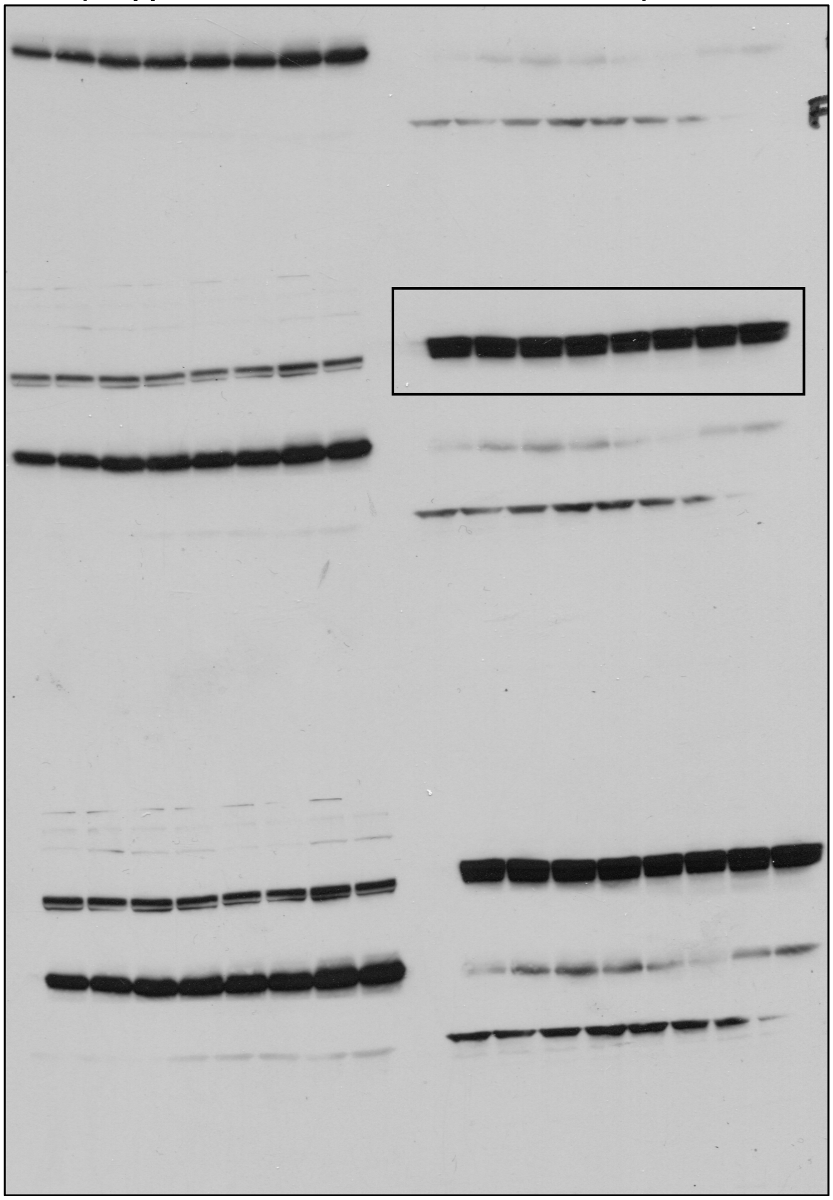

Full unedited images for figure 6E

**V1bR**

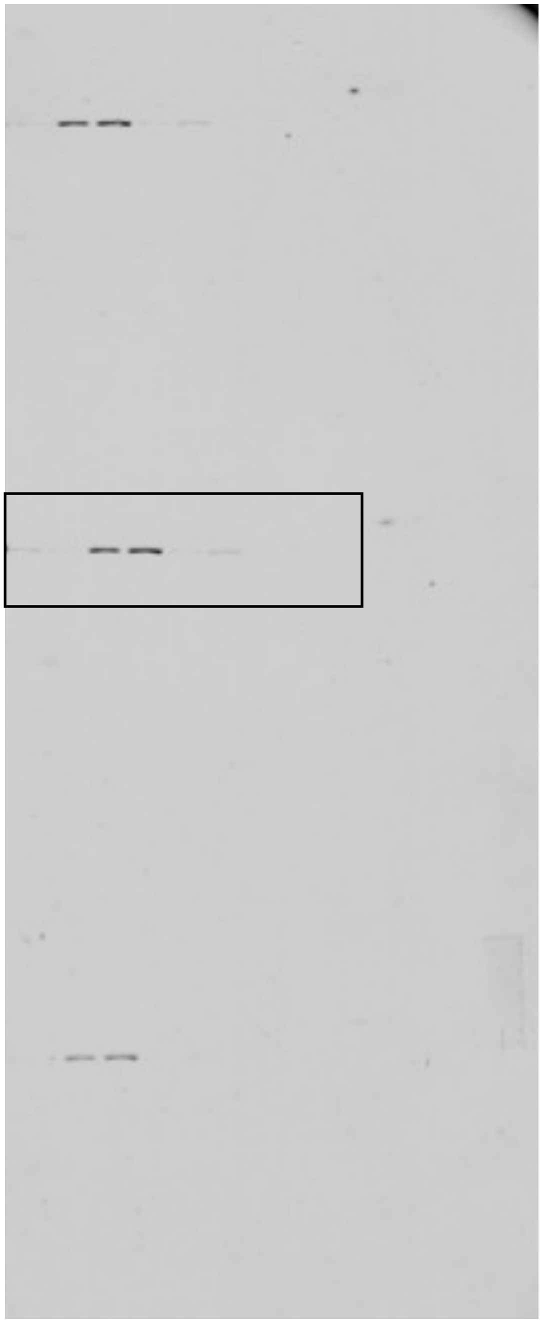

**Actin**

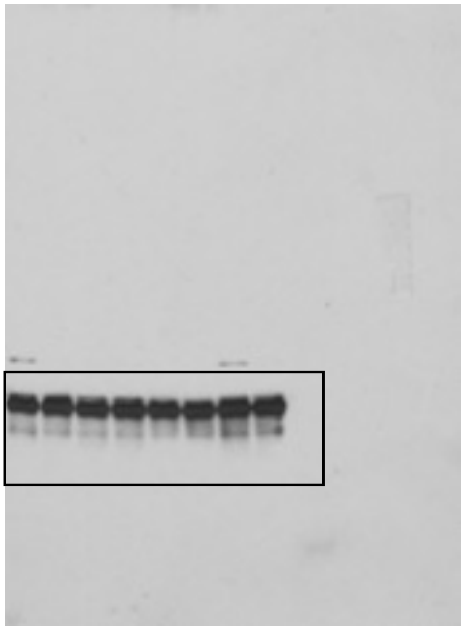

Full unedited images for figure 6F

**KHK**

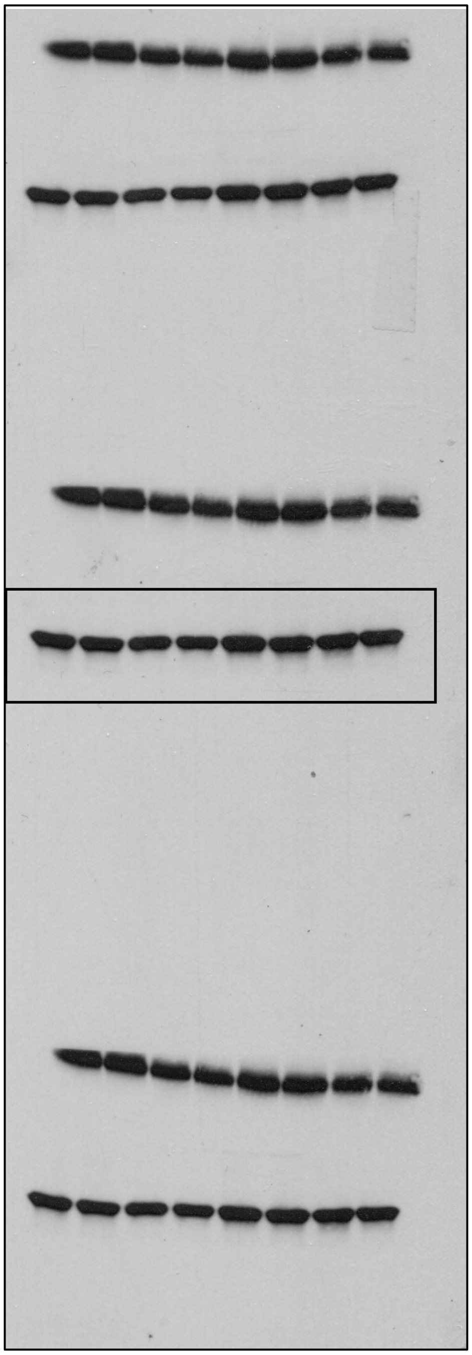

**Actin (stripped and reblotted from KHK)**

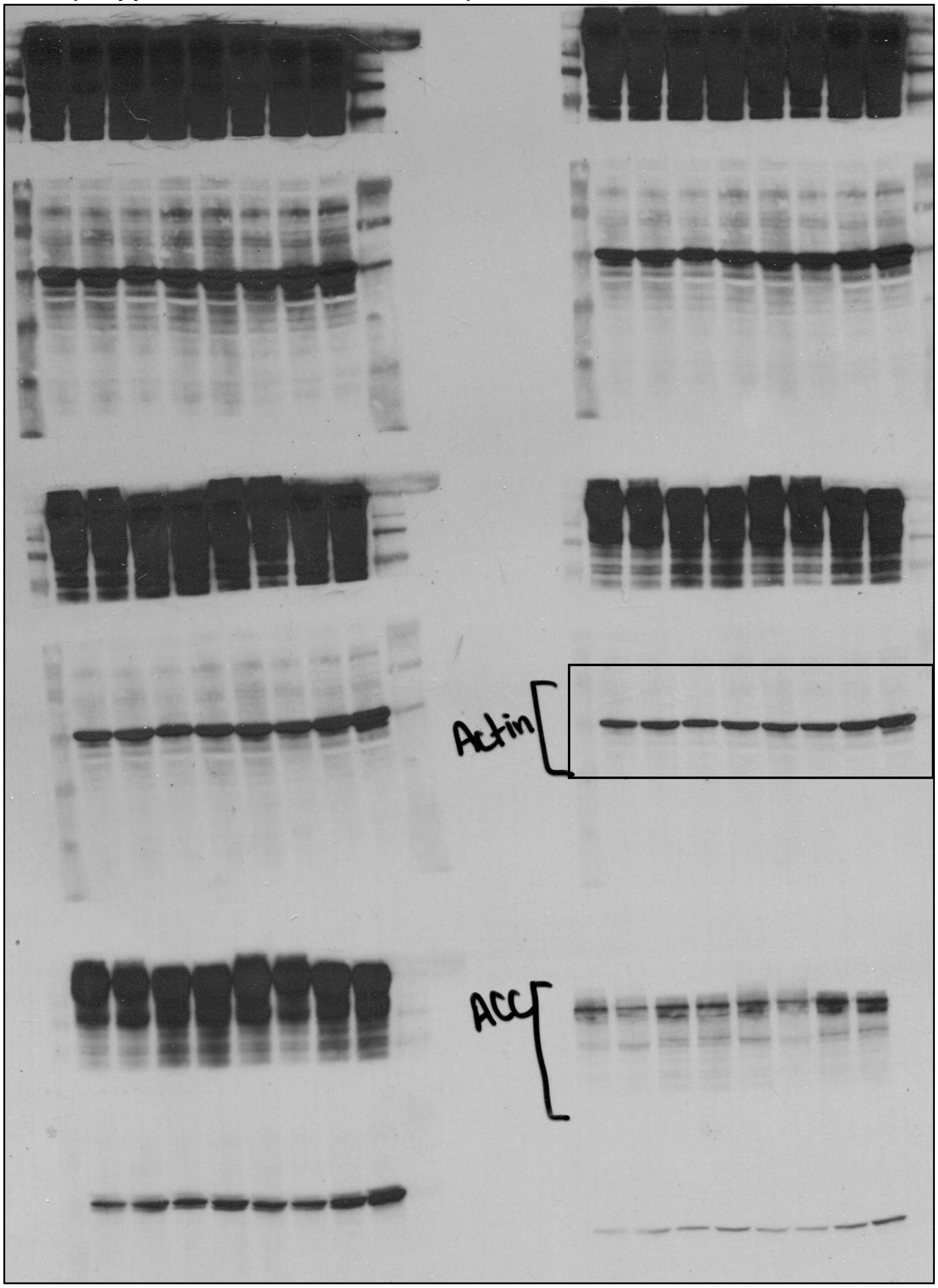

Full unedited images for figure 6G

**FAS**

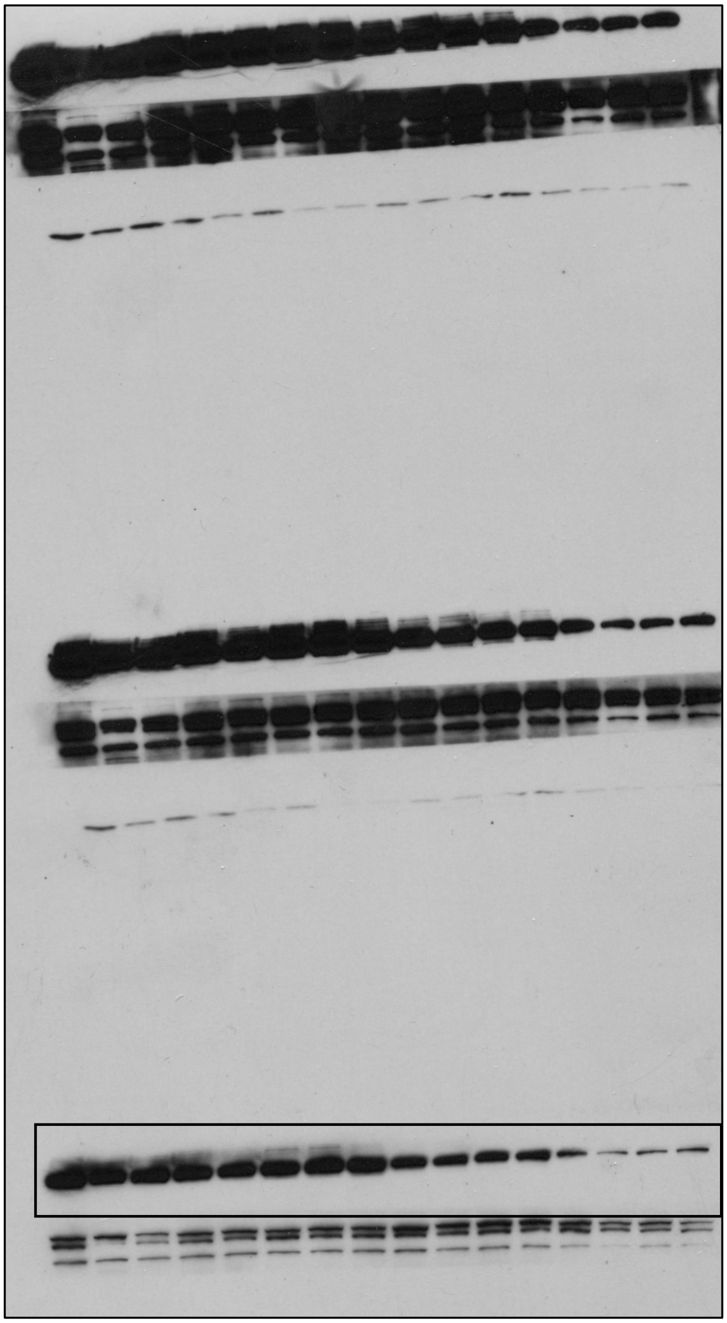

**ACC and Actin**

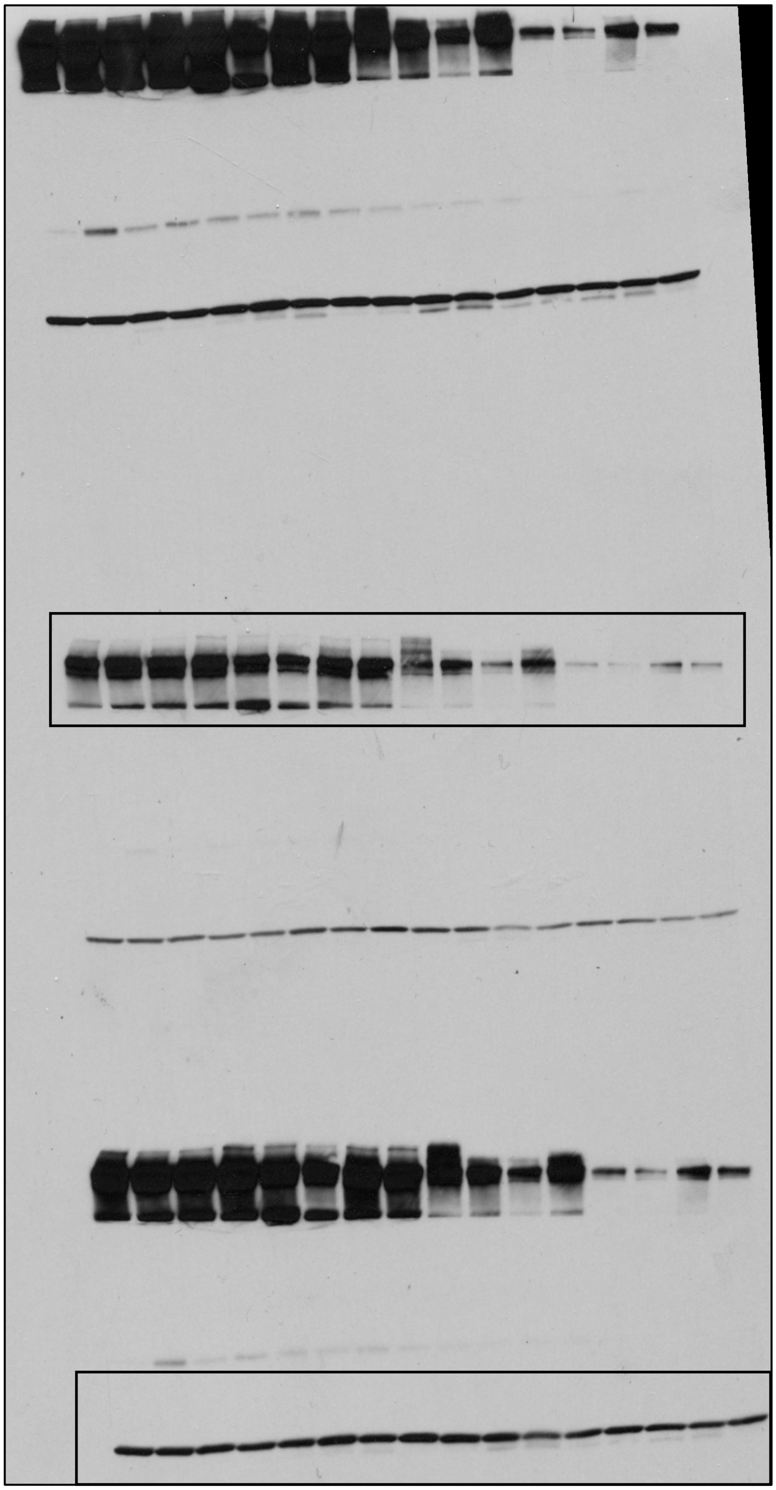

**KHK**

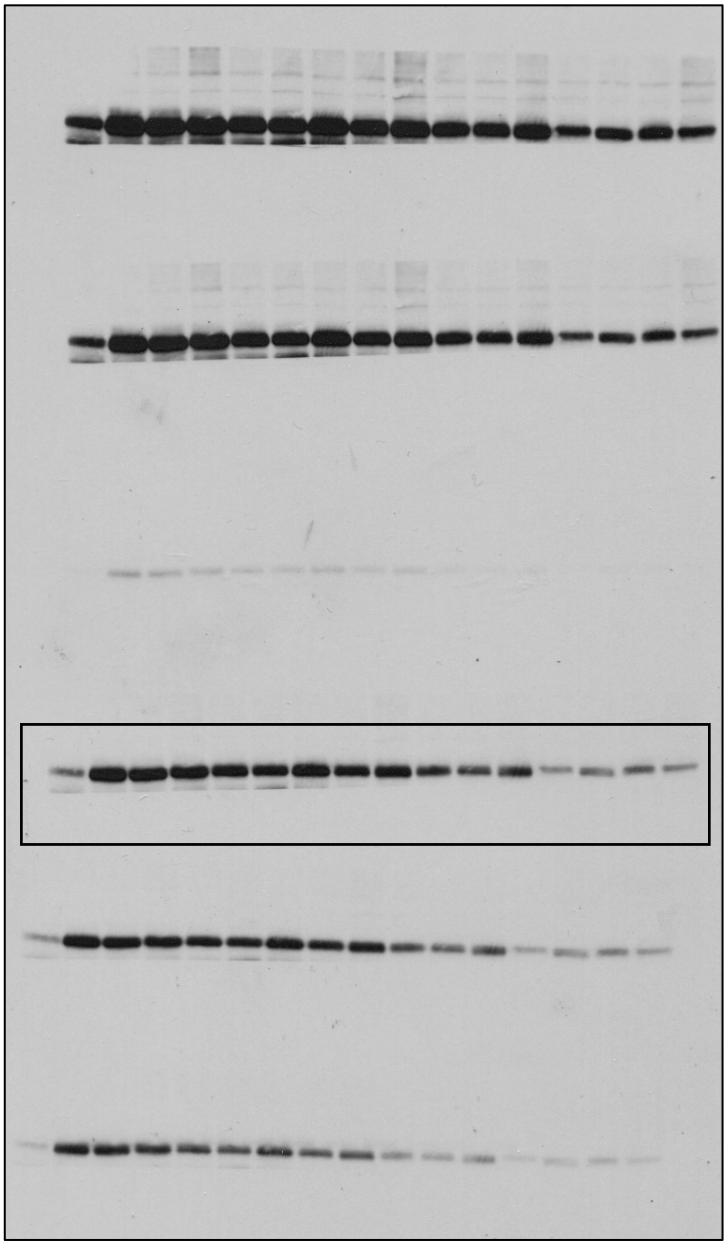

Supplement: Supplemental data [file jciinsight-6-140848-s129.pdf]
